# Examining the association between livestock ownership typologies and child nutrition in the Luangwa Valley, Zambia

**DOI:** 10.1371/journal.pone.0191339

**Published:** 2018-02-06

**Authors:** Sarah E. Dumas, Lea Kassa, Sera L. Young, Alexander J. Travis

**Affiliations:** 1 Master of Public Health program, Cornell University, Ithaca, New York, United States of America; 2 Atkinson Center for a Sustainable Future, Cornell University, Ithaca, New York, United States of America; 3 Baker Institute for Animal Health, Cornell University College of Veterinary Medicine, Ithaca, New York, United States of America; 4 College of Arts and Sciences, Cornell University, Ithaca, New York, United States of America; 5 Department of Population Medicine and Diagnostic Sciences, College of Veterinary Medicine, Cornell University, Ithaca, New York, United States of America; 6 Program in International Nutrition, Cornell University, Ithaca, New York, United States of America; 7 Department of Anthropology, Northwestern University, Evanston, Illinois, United States of America; Indiana University Bloomington, UNITED STATES

## Abstract

**Objective:**

To investigate the association between livestock ownership and dietary diversity, animal-source food consumption, height-for-age z-score, and stunting among children living in wildlife “buffer zones” of Zambia’s Luangwa Valley using a novel livestock typology approach.

**Methods:**

We conducted a cross-sectional study of 838 children aged 6–36 months. Households were categorized into typologies based on the types and numbers of animals owned, ranging from no livestock to large numbers of mixed livestock. We used multilevel mixed-effects linear and logistic regression to examine the association between livestock typologies and four nutrition-related outcomes of interest. Results were compared with analyses using more common binary and count measures of livestock ownership.

**Results:**

No measure of livestock ownership was significantly associated with children’s odds of animal-source food consumption, child height-for-age z-score, or stunting odds. Livestock ownership Type 2 (having a small number of poultry) was surprisingly associated with decreased child dietary diversity (β = -0.477; p<0.01) relative to owning no livestock. Similarly, in comparison models, chicken ownership was negatively associated with dietary diversity (β = -0.320; p<0.01), but increasing numbers of chickens were positively associated with dietary diversity (β = 0.022; p<0.01). Notably, neither child dietary diversity nor animal-source food consumption was significantly associated with height, perhaps due to unusually high prevalences of morbidities.

**Conclusions:**

Our novel typologies methodology allowed for an efficient and a more in-depth examination of the differential impact of livestock ownership patterns compared to typical binary or count measures of livestock ownership. We found that these patterns were not positively associated with child nutrition outcomes in this context. Development and conservation programs focusing on livestock must carefully consider the complex, context-specific relationship between livestock ownership and nutrition outcomes–including how livestock are utilized by the target population–when attempting to use livestock as a means of improving child nutrition.

## Introduction

Nearly 161 million children under the age of five years, or 24.5% of the world’s children, are stunted as a result of chronic undernutrition [1,2]. Stunting is a well documented risk factor for poor motor development, cognitive function, and immune function, increased risk of morbidity and mortality from infectious diseases, and decreased economic productivity in adulthood [1–5]. Almost all stunting occurs in the “first 1000 days” (from conception to two years of age), and its devastating impacts on cognitive and physical development are largely irreversible [1,2].

Stunting has a multifactorial and complex etiology, but its two most important proximate determinants are 1) poor dietary quality among pregnant women, infants, and young children and 2) a high exposure to pathogens causing clinical disease (e.g. diarrhea) or subclinical infection (e.g. environmental enteric dysfunction; [1]). Livestock ownership by low-income rural households can influence both pathways, and the net impact of livestock ownership on stunting may therefore be positive, negative, or neutral, depending on the context.

**Fig 1:**
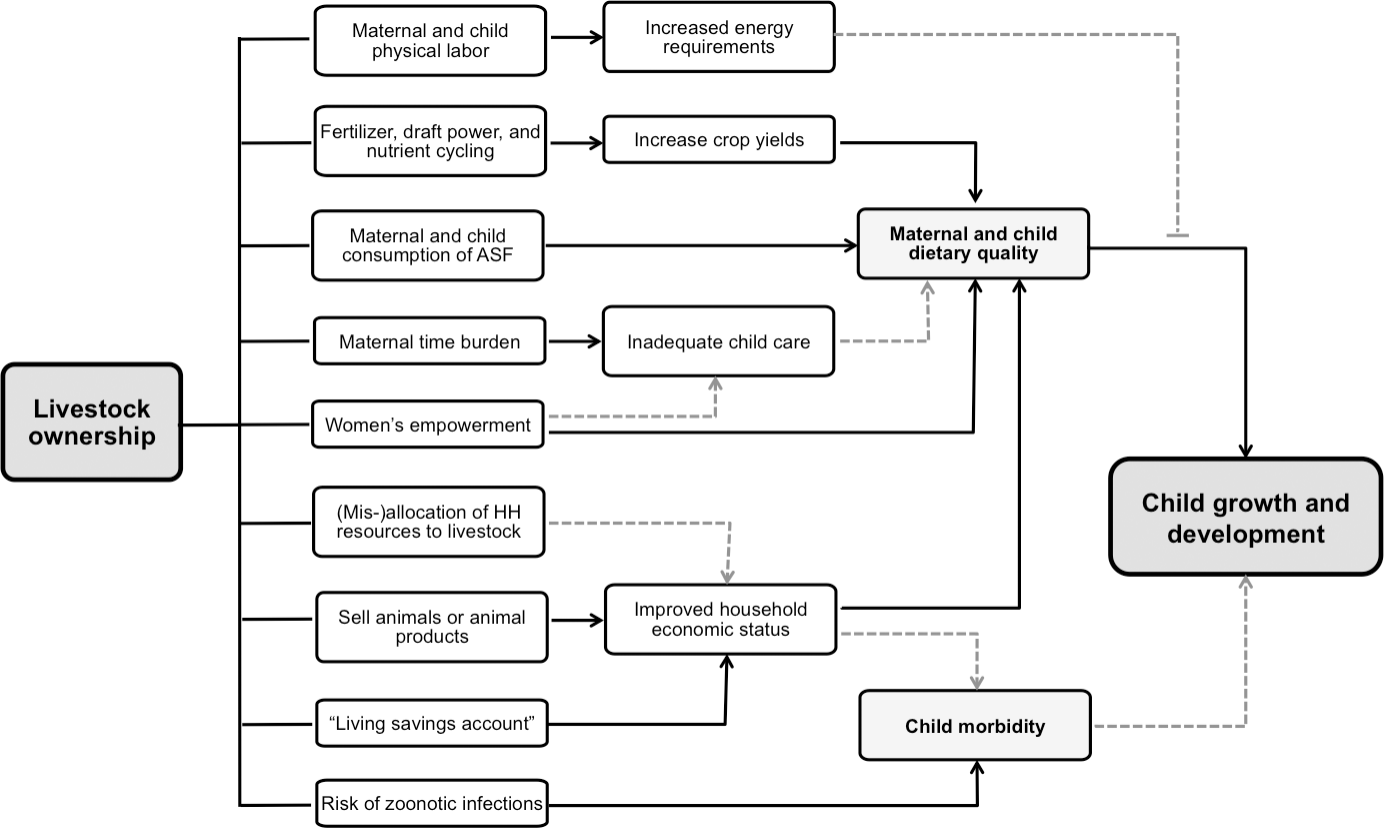
Simplified conceptual framework detailing the key pathways linking livestock ownership to child growth and development. Black solid lines indicate positive influence; grey dashed lines indicate negative influence. Arrows indicate causation; capped lines indicate effect modification.

Livestock production is commonly promoted as a livelihood strategy that can improve children’s access to high-quality animal-source foods (ASF; including meat, milk, and eggs) and increase household incomes. In addition, livestock can positively influence child nutrition through a number of other pathways (Fig 1), including: empowering women; improving crop yields through nutrient cycling, manure fertilizer, and draft power; or as a “living savings account” for storage of capital and consumption smoothing [6–8]. Livestock ownership can also potentially worsen a child’s nutritional status by exposing them to zoonotic pathogens, increasing maternal time burden, competing for household resources, or increasing maternal or child energy demands because of the physical labor required to rear livestock [6,8].

Recent observational research on the impact of livestock managed in traditional extensive systems in sub-Saharan Africa on child stunting has yielded mixed findings (S1 Table, [9–20]), with the most consistent evidence for a positive effect coming from analyses of the specific impact of dairy cow or goat ownership [9–13]. However, others have reported no association between livestock ownership and stunting [14–16], a modest relationship depending on how “livestock ownership” was operationalized [17,18], or even a negative effect in some situations [19,20]. These disparate findings suggest that the link between livestock ownership and child nutrition is complex and context specific, and further research is clearly warranted to better understand this relationship.

One limitation to the existing body of research is the lack of consensus on how to appropriately measure livestock ownership. The most commonly employed measures are a binary indicator of any livestock ownership (e.g. [11,19,20]) or an absolute count of the animals owned (e.g. [12,14,16]). Both methods have clear limitations: a binary indicator of livestock ownership assumes that ownership of one animal has an equal effect on child nutrition as ownership of many animals, while an absolute count of animals assumes that all species and breeds have an equal effect on child nutrition. Both assumptions may be flawed within the borders of our conceptual framework, because the types and numbers of animals that a household owns may affect the amount and frequency of ASF produced for home consumption, the child’s overall exposure to animal feces, the animals’ total contribution to household income or savings, and the amount of household time and labor required.

For example, a household that owns a single village chicken is highly unlikely to slaughter, sell, or eat any of its eggs in the short-term, because the economic incentive is to first allow the flock to grow in order to capitalize on the initial investment of buying that chicken. In contrast, a household owning 30 chickens is able to remove eggs regularly and slaughter or sell chickens as needed without dramatically altering flock dynamics. On the other hand, 30 chickens produce markedly more feces, potentially increasing a child’s risk of diarrheal disease or environmental enteric dysfunction, while a single chicken will likely pose a smaller risk. A binary measure of livestock ownership would treat both households simply as “livestock owners”, missing the fact that they use and benefit from (or are harmed by) their animals in very different ways and to different degrees. Similarly, a single dairy cow can provide daily milk for both sale and home consumption, whereas a single male goat can only be sold or slaughtered once. At the same time, compared to the buck, the dairy cow will require significantly more time and labor to feed and care for it, potentially competing for household resources and maternal time. A count measure of livestock ownership would nonetheless weight each animal equally.

An alternative approach that would capture the differential effects of various types and numbers of livestock would be to use an index, such as the Tropical Livestock Unit (TLU; e.g. [13,18]) score or resale value of the animal [17], to combine a household’s total livestock holdings into a single variable. These methods, however, undervalue small animals and overvalue large animals, which may not be appropriate for assessing the impact on child nutrition outcomes given that small animals can be more readily bartered, sold, or slaughtered to provide food or income on an as-needed basis than can larger, more valuable animals.

There were two main objectives to this research. First, in response to the limitations of existing measures defining livestock ownership, we aimed to develop a new method for differentiating and categorizing types of smallholder livestock ownership for the purposes of examining the livestock-child nutrition link. We have employed a method that combines a household’s TLU with the total number of animals they own to assign them to one of five livestock ownership typologies: no animals of any kind (Type 1); few animals, mostly poultry (Type 2); moderate number of animals, mostly poultry (Type 3); few animals, mixed small and large livestock species (Type 4); and moderate to large number of animals, mixed small and large livestock species (Type 5). This approach assumes that the pattern of livestock ownership (e.g. having a very small flock of chickens, or a moderately sized herd of goats and cattle) is a better proxy measure for how people use their livestock, and that this construct–how people use livestock–is in fact the main determining link between livestock ownership and child nutrition outcomes.

Our second aim was to apply this new measure to an existing dataset to investigate the association between these categories of livestock ownership and child diet and anthropometric status in that context. The Luangwa Valley in Zambia’s Eastern Province presents a unique setting in which to test this methodology and study the link between livestock ownership and child nutrition outcomes. A growing population in the Valley resides within Game Management Areas (“buffer zones”) surrounding national parks and forest reserves, and families rely heavily on the land and natural resources, including wildlife [21]. Although crop farming is the primary income generating activity, yields are inadequate to sustain most households throughout the year [21]. Livestock are therefore an important supplementary livelihood activity for many families and an important potential alternative to unsustainable natural resource use. However, livestock production is constrained by poor forages; minimal access to veterinary care and extension services; wildlife predation; endemic infectious diseases; and indigenous breeds with limited genetic potential for growth and production. For these reasons, livestock ownership is mostly restricted to small numbers of chickens, goats, and pigs raised in traditional scavenging or foraging systems, with chickens being the most commonly owned [22].

In applying the livestock typologies measure to these dataset, we asked three key questions:

1. Is livestock ownership associated with child dietary diversity?
2. Is livestock ownership associated with child ASF consumption?
3. Is livestock ownership associated with child height-for-age z-score (HAZ) or stunting?

Because the livestock typologies measure is new, we also used more conventional measures of livestock ownership (binary measure of any livestock, total counts and counts of individual species, and TLU) to validate our findings. Based on the existing literature and our knowledge of livestock ownership in the region, we hypothesized that livestock ownership would be significantly positively associated with child dietary diversity and ASF consumption, but not HAZ or odds of stunting. This research contributes to the growing body of literature examining the impact of livestock ownership on child nutrition. Using a large sample of young children under 36 months and a unique measure of livestock ownership, we build a greater understanding of the complexities of this relationship in a unique population. As populations expand in similar “buffer zones” around protected areas throughout the world, this study additionally offers insight to how livestock are utilized within this context, with important implications for rural development, public health, and wildlife conservation projects in these areas.

## Methods

### Study area and population

This research took place in 40 rural field sites in Mambwe and Lundazi Districts of Zambia’s Eastern Province, located in four traditionally defined areas (Chiefdoms) in the Luangwa Valley. Sites were purposively selected to take part in a poultry development project by a local non-governmental organization (see “Study context” below). Because villages in the area are very small, multiple villages were included in most field sites (mean 5.6 villages per field site, 222 villages in total). The entire study area is located within the Game Management Areas surrounding four national parks and forest reserves, areas that are home to large populations of wildlife that support a considerable tourism industry.

Although there are limited population data available at the Chiefdom level, Zambia as a whole continues to struggle with poverty, food insecurity, and sub-optimal health, particularly in rural areas. As of the most recent national census, 77.9% of rural households were characterized as in poverty, and 57.7% were in extreme poverty [23]. The HIV epidemic affects 13.3% of Zambian adults [24], while high incidences of malaria, tuberculosis, and maternal, infant, and child morbidities and mortalities strain an overburdened health system [25]. Food security is tenuous for most households, varying dramatically from year to year due to frequent droughts and floods, and smallholder farmers in the Luangwa Valley experience particularly high rates of food insecurity during the lean season, from December to March [21].

### Study context

This is secondary analysis of baseline data collected as part of a larger impact evaluation study being carried out in partnership with Community Markets for Conservation (COMACO; www.itswild.org) [21,22]. The objective of the primary research is to test if an intervention promoting village-scale egg production can improve dietary quality and growth among children 6–36 months of age in participating communities. The data presented here reflect “traditional” livestock ownership practices and dietary behavior and were all collected prior to start of that intervention. The study is registered at ClinicalTrials.gov (ID: NCT02516852); the details will be reported elsewhere after all data are analyzed.

### Data collection

Baseline data were collected over four weeks prior to the intervention, from mid-December 2014 to mid-January 2015. Each field site was marked with a GPS point representing the approximate center of the site. Inclusion criteria for participation were: 1) the household was located ≤ 1.5 km from the field site GPS point; and 2) a child 6–36 months of age lived in the household.

The 20 eligible households nearest to the central GPS point of each field site were recruited and enrolled in the study, for a target of approximately 800 total households. All children 6–36 months of age living within enrolled households were included, and one child from each household was randomly selected during the analysis phase. Individuals underwent a thorough consenting process and the research staff administered in-home questionnaires (available as S1 Appendix) to collect information about household composition, asset ownership, farm production, food security, maternal and child dietary diversity and ASF consumption, child morbidities and breastfeeding history, and subjective maternal wellbeing. Infant and young child feeding practices were measured following WHO recommendations [26]. Child ASF consumption was measured by asking the mother to recall the number of times her child ate meat, fish, *kapenta* (small freshwater fish, usually dried), dairy products, or eggs in the past week. Child morbidity was operationalized as a dichotomous variable, with “morbidity” defined as having any diarrhea, vomiting, fever, or rapid or difficult breathing with coughing in the past 14 days, as observed and recalled by the mother, or malaria diagnosed by a health professional in the past 14 days.

Height and weight measures were then taken on the mother and child using standardized seca 872 electronic scales with mother/child function and seca 213 portable stadiometers (seca GMbH & Co., Hamburg, Germany). For both height and weight, two measures were taken; a third measure was taken if there was a difference of at least 0.5 kg or 1.0 cm between the first two measures [27]. The mean of the two most similar measures was defined as the child’s height and weight. The entire procedure, including questionnaires and anthropometry, lasted approximately 45 minutes per household.

### Variable definitions

#### Exposure variables

Households were assigned to one of five “livestock ownership typologies” based on the types and numbers of livestock they owned. To create this typology, we generated two standard measures: 1) total number of animals owned, where all species are equally weighted; and, 2) a TLU score, which uses a weighted value for each species to estimate the total value of their livestock holdings. The TLU weighting factors used were 0.70 for cattle, 0.20 for pigs, 0.10 for sheep and goats, 0.02 for ducks and guinea fowl, 0.01 for chickens, and 0.005 for pigeons [28]. Then, each variable was categorized into tertiles, and the two categorical variables were cross-tabulated, revealing five distinct patterns, or typologies, of livestock ownership (Tables 1 and 2). For comparison, we additionally considered eleven other measures of livestock ownership: binary measure of any livestock ownership; total number of animals owned; TLU owned; binary measures of any chickens, any goats, any pigs, and any cattle; and individual counts of the number of chickens, goats, pigs, and cattle.

**Table 1:** Five patterns of livestock ownership, or typologies, were defined by the total number of livestock and tropical livestock units (TLU) owned by household.

		TLU, tertiles			
		1	2	3	Total number of households
**Total number of livestock, tertiles**	**1**	**n = 309**	0	0	309
	**2**	0	**n = 196**	**n = 54**	250
	**3**	0	**n = 62**	**n = 217**	279
Total number of households		309	258	271	**838**

**Table 2:** Characteristics of the five livestock ownership typologies.

	Type 1 (n = 309)	Type 2 (n = 196)	Type 3 (n = 62)	Type 4 (n = 54)	Type 5 (n = 217)
Description	No livestock of any kind	Few animals, mostly poultry (e.g. 4 chickens)	Moderate number of animals, mostly poultry (e.g. 10 chickens)	Few animals, mixed large and small livestock (e.g. 2 goats, 2 pigs, 7 chickens)	Many animals, mixed large and small livestock (e.g. 2 cattle, 2 sheep, 15 chickens)
**Mean TLU (range)**		0.04 (0.01–0.11)	0.1 (0.09–0.12)	0.67 (0.14–3.50)	1.75 (0.13–20.08)
**Mean no. of animals (range)**		4.22 (1–8)	10.56 (9–14)	4.83 (1–8)	22.51(9–119)
**Own chickens (%)**		96.9%	100.0%	40.7%	94.0%
**No. of chickens, mean (range)**		4.1 (0–8)	10.1 (6–12)	1.4(0–7)	13.3 (0–50)
**Own goats (%)**		1.0%	0.0%	40.7%	33.6%
**No. of goats, mean (range)**		0.01 (0–1)	0.0	1.3(0–7)	2.2(0–42)
**Own pigs (%)**		0.0%	0.0%	53.7%	30.9%
**No. of pigs, mean (range)**		0.0	0.0	1.6(0–7)	2.7(0–42)
**Own cattle (%)**		0.0%	0.0%	13.0%	21.7%
**No. of cattle, mean (range)**		0.0	0.0	0.3(0–5)	1.1(0–15)
**Own ducks (%)**		5.1%	1.6%	0.0%	12.9%
**No. of ducks, mean (range)**		0.1(0–5)	0.0 (0–3)	0.0	0.7(0–11)
**Own pigeons (%)**		0.5%	8.1%	0.0%	6.0%
**No. of pigeons, mean (range)**		0.02(0–3)	0.5(0–8)	0.0	1.7(0–80)
**Own guinea fowl (%)**		1.0%	0.0%	1.9%	6.0%
**No. of guinea fowl, mean (range)**		0.02(0–2)	0.0	0.1(0–7)	0.4(0–26)
**Own sheep (%)**		0.0%	0.0%	5.6%	4.1%
**No. of sheep, mean (range)**		0.0	0.0	0.2 (0–4)	0.4(0–33)

#### Outcome variables

The child’s dietary quality was assessed with two measures: 1) individual dietary diversity score (DDS), the number of food groups out of seven consumed by the child in the 24 hours prior to the survey [26]; and 2) a dichotomous indicator that the child consumed any ASF over the past 7 days. Nutritional status was assessed by child HAZ, where the reference population is based on the WHO Child Growth Standards. Children with HAZ < -2 were classified as stunted, and outliers with HAZ values > +6 or < -6 were excluded during data cleaning as biologically implausible (n = 3) [29].

#### Control and descriptive variables

Because livestock are often used in rural areas as an instrument for wealth storage, household wealth was controlled for in all models to eliminate the concern that any association between livestock ownership and child diets or nutrition represented a general effect of wealth, rather than a specific effect of livestock. Wealth was assessed with an asset index generated using principal components analysis based on indicators for household dwelling quality and size, electricity access, use of paid agricultural labor, and ownership of various household assets (TV, radio, CD or DVD player, bicycle, mobile phone, plough, mattress, bed, sofa, table, solar panel, battery, bank account). Livestock ownership was not included. The first component (eigenvalue = 5.830, explaining 29.2% of the variability in the sample, Cronbach’s alpha = 0.857) was retained and used to generate a tertile measure of wealth.

Additional covariates were household size; sex of head of household and whether he or she had completed primary school; maternal age, height, and body mass index (BMI); and child sex, age, and breastfeeding history (binary indicator of whether they were exclusively breastfed to 6 months of age based on the mother’s response to eight questions about the timing of her initiation, duration, and cessation of breastfeeding and the introduction of water and solid foods). Household COMACO membership status was initially included in all models but was highly non-significant and was not included in final models. Similarly, distance from the central GPS point was considered as a potential confounder on the assumption that more remote households maybe be systematically worse off than those more centrally located. However, distance from the central GPS point was not significantly correlated with the asset index or household food insecurity. Given this, along with the fact that each field site was composed of multiple villages, distance from the GPS point was not included in any final models. Household food insecurity over the one month prior to the survey was assessed by the Household Food Insecurity Access Scale (HFIAS; [30]), retained for descriptive purposes as a continuous variable from 0 (completely food secure) to 27 (severely food insecure) and categorized as food security (HFIAS = 0), mildly food insecurity (HFIAS = 1–9), moderately food insecure (HFIAS = 10–18), or severely food insecure (HFIAS = 19–27).

### Data handling

Data were collected on handheld Android mobile devices (Samsung Galaxy Tab 4 7.0, Samsung Electronics Co., Suwon, South Korea) using ODK Collect (Open Data Kit, https://opendatakit.org/). Data were pulled daily from the tablets using ODK Briefcase and stored on a password-secured local server. Data cleaning and analysis were completed in Stata (Stata/IC version 14.0, StataCorp, College Station, Texas).

### Analytical methods

Descriptive analysis of all variables was first performed to better understand the characteristics of the study population. Bivariate analyses (chi-squared and ANOVA) of the association between measures of livestock ownership and measures of child DDS, ASF consumption, and stunting status were performed. Associations were considered significant at p <0.05.

To further examine the association between livestock ownership and continuous outcomes of interest (DDS and HAZ), we fitted multi-level mixed effect models with field site random effects nested within Chiefdom to account for potential clustering of outcomes within communities. To examine the association between livestock ownership and binary outcomes of interest (any ASF consumption and stunting), we fitted generalized linear mixed effect models (GLMM) with a binomial family and logit-link function, again with field site random-effects nested within Chiefdom. All models included controls for household characteristics (household size, wealth, sex of head of household and whether they completed primary school), maternal age, child characteristics (sex, age, and age squared). Models for HAZ and stunting additionally included control variables for maternal BMI and height, child breastfeeding history, and recent child history of any morbidity. Covariates were selected a priori based on the literature.

### Ethical Standards Disclosure

All procedures, protocols, and research materials underwent an internal review process at COMACO and were approved by the Institutional Review Board at Cornell University (Protocol ID#: 1402004456). In the field, approval was first obtained from Senior Chief Nsefu and Chiefs Mnkhanya, Jumbe, and Mwanya, who granted permission for all field activities in their respective Chiefdoms. We then met individually with key Village Headmen from selected field sites to inform them of our activities and obtain their support. At the time of enrollment, all participants provided individual written informed consent; separate consents were obtained for the household interview, the maternal interview, and parental consent for anthropometric measurements. In the case of an illiterate participant, the interviewer read the consent forms in full, took a thumbprint from the participant, and acquired a witness signature confirming that informed consent was appropriately obtained.

## Results

Of the 838 eligible children with complete dietary recall and livestock ownership data, biologically plausible anthropometric data were available for 835 of these children. Despite record high cereal production in 2014 [31], food insecurity was prevalent at the time of data collection (the start of the lean season, when the previous year’s harvest has largely been completely consumed, but new crops have not yet reached harvest), with 43.7% of households reporting mild food insecurity and 41.1% reporting moderate or severe food insecurity over the past 30 days (Table 3). In an unadjusted analysis, household food insecurity status was not significantly associated with children’s DDS (p = 0.743). In contrast, compared to children living in food secure households (HFIAS = 0), severe food insecurity (HFIAS >19) was associated with significantly lower odds of ASF consumption (OR = 0.37, p = 0.028). Notably, only 31 children (3.5% of the sample) lived in severely food insecure households. Compared to children living in food secure households, children living in mildly or moderately food insecure households did not have significantly different odds of ASF consumption (p = 0.805 and p = 0.110, respectively).

**Table 3:** Characteristics of participating households and children (n = 838).

**Household characteristics**	
Household size, mean (SD)	5.3 (2.2)
Number of children under 5 years, mean (SD)	1.5 (0.7)
Head of household age, mean years (SD)	34.6 (9.9)
Head of household sex, % female	20.6
Head of household education, % completing primary	57.5
Electricity access, %	27.7
Protected water source, %	79.1
Thatch roofing on house, %	64.0
Mud flooring in house, %	85.9
Latrine type in household	
None	2.3
Shared pit latrine*	50.8
Private pit latrine*	46.9
Household Food Insecurity Access Scale, mean (SD)	8.2 (5.9)
Food secure (HFIAS = 0), %	15.2
Mildly food insecure (HFIAS = 1–9), %	43.7
Moderately food insecurity (HFIAS = 10–18), %	37.6
Severely food insecure (HFIAS = 19–27), %	3.5
**Child characteristics**	
Age, mean months (SD)	21.2 (8.6)
Sex, % female	53.2
Ever breastfed, %	88.2
Exclusively breastfed to 6 months, %	54.3
Currently breastfeeding, %	45.2
Fever in the past 14 days, %	55.4
Diarrhea in the past 14 days, %	45.2
Acute respiratory illness in the past 14 days, %	24.3
≥ 1 morbidity in the past 14 days, %	75.4
Dietary diversity score, mean out of seven (SD)	3.4 (1.5)
Minimum dietary diversity met (≥ 4 food groups eaten), %	41.1
Minimum acceptable diet met (children 6-24mo), %	18.4
Any ASF consumption in the past 7d, %	81.6
Frequency of ASF consumption in past 7d, mean (SD)	5.2 (4.8)
HAZ, mean (SD)	-1.65 (1.38)
Stunted (HAZ< -2), %	40.0
Severely stunted (HAZ< -3), %	13.9

ASF, animal source foods; HAZ, height-for-age z-score

* Traditional pit latrine. There were no Ventilated Improved Pit (VIP) latrines, pit latrines with slabs, or otherwise improved latrines.

Mean child DDS was low, with less than half of children having consumed a minimally diverse diet the day before the survey. While the majority of children had been breastfed at some point in their lifetime, just over half were exclusively breastfed to 6 months of age. Three quarters of women reported that their child experienced at least one morbidity in the 14 days prior to the survey, with the majority experiencing multiple morbidities. Prevalence of stunting and severe stunting was very high (Table 3).

**Fig 2:**
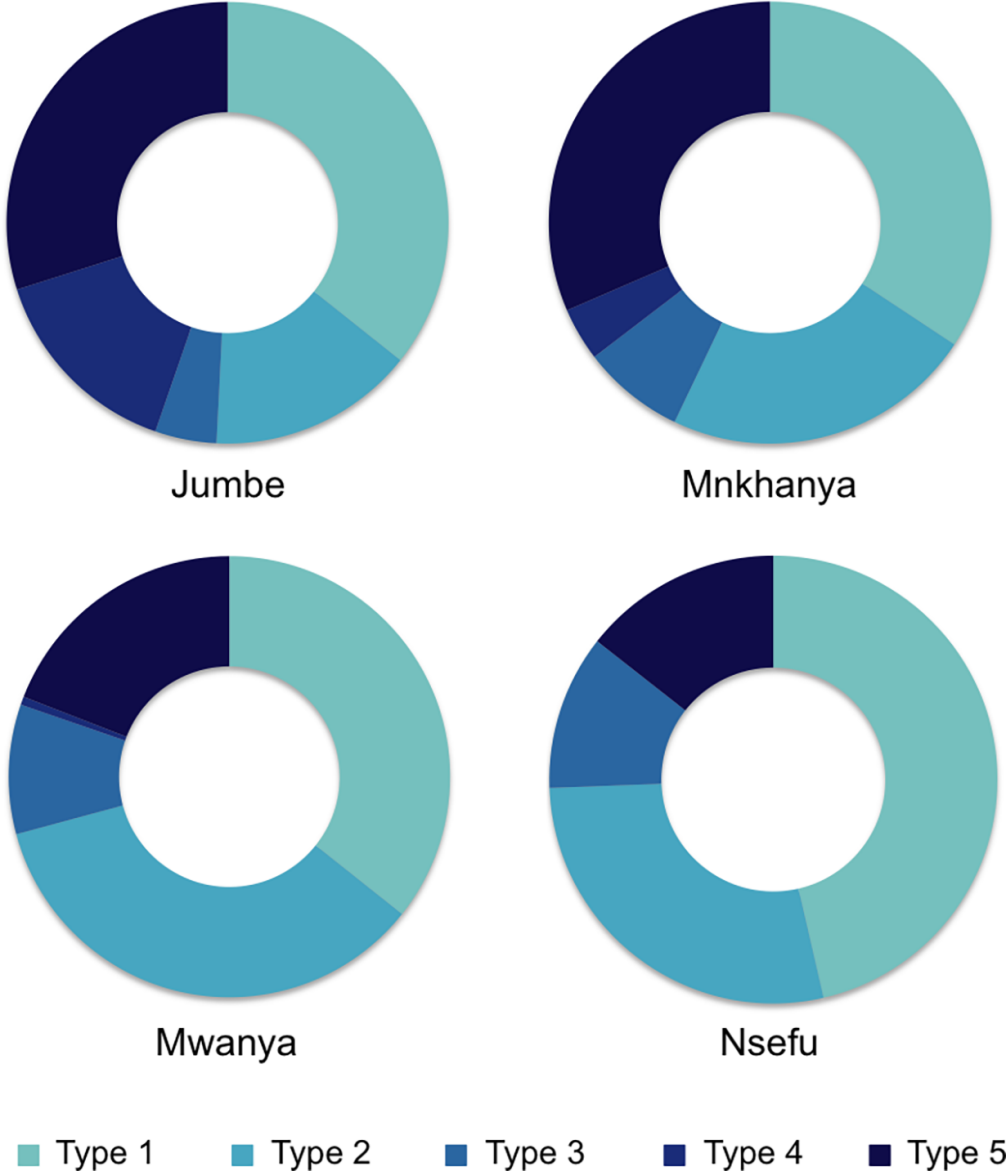
The distribution of livestock typologies in the four Chiefdoms. Typologies were defined as follows: no animals of any kind (Type 1); few animals, mostly poultry (Type 2); moderate number of animals, mostly poultry (Type 3); few animals, mixed small and large livestock species (Type 4); and moderate to large number of animals, mixed small and large livestock species (Type 5).

Overall, 63.1% of households owned livestock of some kind, with chickens being the most commonly owned, followed by goats and pigs (Table 4). Despite widespread ownership of at least one animal, total livestock holdings were small, with a median TLU of just 0.05, the equivalent of five chickens. Livestock ownership patterns varied significantly by Chiefdom (p< 0.001; Fig 2). Mwanya and Nsefu Chiefdoms, in particular, had a high number of households categorized as Type 1 or Type 2 typologies, while Jumbe and Mnkhanya Chiefdoms had a high number of households categorized as Type 4 or Type 5.

**Table 4:** Unadjusted associations between various measures of livestock ownership and stunting, dietary diversity, or animal source food consumption.

Variable	Overall	Not Stunted†	Stunted†	p-value	Low DDS‡	High DDS‡	p-value	No ASF^§^	Any ASF^§^	p-value
Children, n	838	501	334		494	344		154	684	
Ownership of any livestock, %	63.1%	65.7%	59.6%	0.074	63.2%	63.1%	0.982	55.8%	64.8%	**0.038**
Chicken	57.0%	59.1%	54.2%	0.162	57.5%	56.4%	0.753	49.4%	58.8%	**0.033**
Goat	11.6%	11.4%	12.0%	0.792	12.1%	10.8%	0.537	11.0%	11.7%	0.818
Pig	11.5%	11.8%	11.1%	0.757	11.9%	10.8%	0.596	11.0%	11.5%	0.858
Cattle	6.4%	6.4%	6.3%	0.954	6.3%	6.7%	0.812	3.9%	7.0%	0.154
Ducks	4.7%	5.4%	3.6%	0.229	4.9%	4.4%	0.737	3.9%	4.8%	0.622
Pigeon	2.3%	2.6%	1.8%	0.449	2.2%	2.3%	0.925	0.0%	2.8%	**0.037**
Guinea fowl	1.9%	1.4%	2.7%	0.181	1.8%	2.0%	0.825	0.0%	2.3%	0.055
Sheep	1.4%	1.2%	1.5%	0.711	1.6%	1.2%	0.585	1.3%	1.5%	0.878
Total number of animals owned, mean (SD)	7.91 (12.12)	8.05 (11.36)	7.70 (13.21)	0.684	8.01 (13.66)	7.77 (9.50)	0.778	5.85 (11.95)	8.37 (12.12)	**0.020**
TLU, mean (SD)	0.51 (1.63)	0.50 (1.49)	0.54 (1.81)	0.733	0.53 (1.82)	0.49 (1.29)	0.742	0.42 (1.85)	0.54 (1.57)	0.403
Livestock Typology^#^										
Type 1	36.9%	34.3%	40.4%	0.194	36.8%	36.9%	0.301	44.2%	35.2%	**0.018**
Type 2	23.4%	25.2%	21.0%		25.3%	20.6%		22.1%	23.7%	
Type 3	7.4%	8.4%	6.0%		6.1%	9.3%		7.1%	7.5%	
Type 4	6.4%	5.8%	7.5%		6.3%	6.7%		9.7%	5.7%	
Type 5	25.9%	26.4%	25.2%		25.5%	26.5%		16.9%	27.9%	

DDS, dietary diversity score; ASF, animal source foods; TLU, Tropical Livestock Units

†Stunted is defined as a height-for-age z-score of < -2.

‡Low DD is defined as eating 0–3 out of 7 food groups in the 24 hours preceding the survey; high DD is eating 4–7 food groups.

§ASF consumption was operationalized as a dichotomous variable indicating that a child had any amount of meat, fish, dairy, or eggs in the past 7 days

^#^Hierarchical livestock typology: Type 1 = no livestock; Type 2 = few poultry; Type 3 = many poultry; Type 4 = few mixed livestock; Type 5 = many mixed livestock.

No measure of livestock ownership was significantly associated with stunting or meeting the minimum DDS in unadjusted t-tests or chi-squared tests (Table 4). There was a marginally lower prevalence of any livestock ownership among households where the index child was stunted, but this difference was not statistically significant. In unadjusted comparisons, chicken, pigeon, and any livestock ownership were significantly associated with ASF consumption (Table 4). The chi-squared test indicates that a child’s ASF consumption is not independent of their household’s livestock ownership typology (p = 0.018). In particular, compared to children consuming any ASF over the past 7 days, children who consumed no ASF were more likely to live in Type 1 (no livestock) households and less likely to live in a Type 5 (many animals of high value) households.

### Is livestock ownership associated with child dietary diversity?

In the multi-level mixed effect model, livestock ownership Type 2 was associated with significantly lower DDS among children (Table 5 and S3 Table). The analysis was followed by a post-hoc pairwise comparison among the five levels of livestock typology using a Sidak correction for multiple comparisons; DDS was only significantly different between children living in households with livestock ownership Type 1 and Type 2 (β = -0.50, p = 0.002, with children living in Type 2 households having lower DDS). Living in Mwanya was associated with significantly higher DDS. Education of the head of household, wealth, maternal age, and child age were strongly predictive of increased DDS, while household size was strongly predictive of decreased DDS. A recent history of illness was positively predictive of DDS, which may reflect a local practice of giving children raw eggs with traditional medicines, though further investigation of this idea is beyond the scope of this paper.

**Table 5:** Summary of multi-level mixed effect model (maximum likelihood estimates) assessing the effect of livestock ownership typology on child dietary diversity (n = 811)^†^.

	Adjusted regression coefficient (95% CI)	P-value
Livestock ownership typology‡ (vs. Type 1)		
Type 2	-0.460 (-0.716, -0.204)	**<0.001**
Type 3	-0.206 (-0.600, 0.187)	0.306
Type 4	-0.326 (-0.750, 0.099)	0.132
Type 5	-0.150 (-0.418, 0.118)	0.273
Household size	-0.065 (-0.114, -0.015)	**0.010**
Female head of household	-0.239 (-0.493, 0.015)	0.065
Head of household completed primary education	0.273 (0.071, 0.475)	**0.008**
SES tertile		
Medium vs. low	0.299 (0.064, 0.535)	**0.013**
High vs. low	0.374 (0.122, 0.627)	**0.004**
Maternal age, years	0.019 (0.005, 0.032)	**0.005**
Female child	-0.033 (-0.222, 0.156)	0.733
Child age, months	0.180 (0.121, 0.238)	**<0.001**
Child age, months, squared	-0.003 (-0.004, -0.002)	**<0.001**
History of morbidity, past 14d	0.235 (0.009, 0.461)	**0.042**
Chiefdom (vs. Jumbe)		
Mnkhanya	0.126 (-0.144, 0.395)	0.361
Mwanya	0.348 (0.030, 0.665)	**0.032**
Nsefu	-0.142 (-0.483, 0.199)	0.413
*Between household (Level 1) variance*	*1*.*823*	
*Between village (Level 2) variance*	*0*.*029*	
*ICC*	*0*.*016*	
*Overall R*^*2*^	*0*.*126*	

SES, socioeconomic status; ICC, intraclass correlation coefficient

† Model includes fixed effects of Chiefdom and random effect of field site (village).

‡ Hierarchical livestock typology: Type 1 = no livestock; Type 2 = few poultry; Type 3 = many poultry; Type 4 = few mixed livestock; Type 5 = many mixed livestock

### Is livestock ownership associated with child ASF consumption?

In generalized linear mixed effect models, no livestock typology was significantly associated with odds of child ASF consumption, although Type 5 typology approached significance (Table 6 and S3 Table). In a post-hoc pairwise comparison among Chiefdoms, the odds of any ASF consumption were significantly higher among children in Mwanya versus those in Mnkhanya (OR = 3.61, p = 0.016) and Nsefu (OR = 4.95, p = 0.004). The highest tertile of household wealth and child age were the only other significant predictors of child ASF consumption. In the initial models, a recent history of morbidity was considered as a potential predictor of child ASF consumption; however, it was not significant and did not meaningfully affect the point estimates for the other predictors, and it was therefore dropped from the final regression in order to retain the most parsimonious model.

**Table 6:** Summary of generalized linear mixed effect model (maximum likelihood estimates) assessing the effects of livestock ownership typology on odds of any ASF consumption in the past 7 days (n = 812)^†^.

	Adjusted odds ratio (95% CI)	P-value
Livestock ownership typology‡ (vs. Type 1)		
Type 2	0.976 (0.582, 1.636)	0.927
Type 3	0.932 (0.419, 2.075)	0.864
Type 4	0.682 (0.313, 1.488)	0.336
Type 5	1.782 (0.990, 3.207)	0.054
Household size	0.975 (0.879, 1.083)	0.639
Female head of household	1.011 (0.595, 1.719)	0.966
Head of household completed primary education	1.245 (0.818, 1.894)	0.307
SES tertile		
Medium vs. low	1.348 (0.847, 2.145)	0.208
High vs. low	2.009 (1.183, 3.411)	**0.010**
Maternal age, years	1.020 (0.991, 1.050)	0.139
Female child	1.001 (0.678, 1.478)	0.980
Child age, months	1.347 (1.203, 1.508)	**<0.001**
Child age, months, squared	0.994 (0.991, 0.997)	**<0.001**
Chiefdom (vs. Jumbe)		
Mnkhanya	0.689 (0.387, 1.227)	0.206
Mwanya	2.706 (1.186, 6.174)	**0.018**
Nsefu	0.488 (0.245, 0.972)	**0.041**
*Between village (Level 2) variance*	*0*.*176*	
*ICC*	*0*.*051*	
*Overall R*^*2*^	*0*.*138*	

SES, socioeconomic status; ICC, intraclass correlation coefficient

† Model includes fixed effects of Chiefdom and random effect of field site (village).

‡ Hierarchical livestock typology: Type 1 = no livestock; Type 2 = few poultry; Type 3 = many poultry; Type 4 = few mixed livestock; Type 5 = many mixed livestock

### Is livestock ownership associated with child HAZ or stunting?

Livestock ownership typology was not significantly associated with child HAZ (Table 7 and S3 Table) or stunting odds (Table 8 and S3 Table). In both models, maternal BMI, maternal height, and female sex of the child were strongly associated with higher HAZ and decreased odds of stunting, while child age was strongly associated with lower HAZ and increased odds of stunting. Wealth was not associated with HAZ or stunting odds. Despite differences in DDS and ASF consumption by Chiefdom, there was no difference in mean HAZ or stunting odds across Chiefdoms. Although no households in the sample had an improved sanitation facility, having a private (vs. shared) latrine was included in initial models for both HAZ and stunting outcomes but was associated with high p-values (0.618 and 0.970, respectively) and had no effect on the estimates or p-values of other covariates in the model. It was therefore not included in the final models based on a tenuous theoretical connection between private sanitation facilities and child nutrition outcomes.

**Table 7:** Summary of multi-level mixed effect model (maximum likelihood estimates) assessing the effect of livestock typology on child height-for-age z-score (n = 799)^†^.

	Adjusted regression coefficient (95% CI)	P-value
Livestock ownership typology‡ (vs. Type 1)		
Type 2	0.206 (-0.025, 0.438)	0.080
Type 3	0.036 (-0.315, 0.388)	0.840
Type 4	-0.326 (-0.705, 0.052)	0.091
Type 5	-0.032 (-0.274, 0.211)	0.798
Household size	-0.017 (-0.061, 0.028)	0.460
Female head of household	-0.030 (-0.258, 0.197)	0.793
Head of household completed primary education	-0.013 (-0.193, 0.169)	0.893
SES tertile		
Medium vs. low	0.190 (-0.021, 0.401	0.078
High vs. low	0.155 (-0.071, 0.380)	0.179
Maternal age, years	0.000 (-0.012, 0.012)	0.942
Maternal BMI	0.043 (0.015, 0.072)	**0.003**
Maternal height, cm	0.052 (0.038, 0.067)	**<0.001**
Female child	0.339 (0.169, 0.508)	**<0.001**
Child age, months	-0.122 (-0.175, -0.069)	**<0.001**
Child age, months, squared	0.002 (0.001, 0.003)	**<0.001**
Child exclusively breastfed to 6mo	-0.039 (-0.214, 0.139)	0.664
History of any morbidity, past 14d	-0.102 (-0.307, 0.101)	0.327
Any ASF consumed in past 7d	-0.038 (-0.265, 0.189)	0.743
Chiefdom (vs. Jumbe)		
Mnkhanya	-0.165 (-0.378, 0.047)	0.130
Mwanya	-0.179 (-0.433, 0.076)	0.158
Nsefu	-0.095 (-0.365, 0.175)	0.507
*Between household (Level 1) variance*	*1*.*452*	
*Between village (Level 2) variance*	*0*.*000*	
*ICC*	*0*.*000*	
*Overall R*^*2*^	*0*.*140*	

SES, socioeconomic status; BMI, body mass index; ASF, animal source foods; ICC, intraclass correlation coefficient

† Model includes fixed effects of Chiefdom and random effect of field site (village).

‡ Hierarchical livestock typology: Type 1 = no livestock; Type 2 = few poultry; Type 3 = many poultry; Type 4 = few mixed livestock; Type 5 = many mixed livestock

**Table 8:** Summary of generalized linear mixed effect model (maximum likelihood estimates) assessing the effects of livestock ownership typology on odds of child stunting (n = 804)^†^.

	Adjusted odds ratio (95% CI)		P-value
Livestock ownership typology‡ (vs. Type 1)			
Type 2	0.682 (0.444, 1.046)		0.080
Type 3	0.751 (0.386, 1.460)		0.399
Type 4	1.411 (0.712, 2.798)		0.324
Type 5	1.019 (0.651, 1.597)		0.934
Household size	1.061 (0.976, 1.152)		0.163
Female head of household	0.870 (0.571, 1.325)		0.516
Head of household completed primary education	1.129 (0.808, 1.579)		0.476
SES tertile			
Medium vs. low	0.806 (0.547, 1.191)		0.280
High vs. low	0.818 (0.540, 1.242)		0.346
Maternal age, years	0.986 (0.965, 1.001)		0.222
Maternal BMI	0.935 (0.886, 0.987)		**0.015**
Maternal height, cm	0.900 (0.873, 0.927)		**<0.001**
Female child	0.619 (0.453, 0.849)		**0.003**
Child age, months	1.232 (1.111, 1.367)		**<0.001**
Child age, months, squared	0.996 (0.994, 0.998)		**0.001**
Child exclusively breastfed to 6mo	0.885 (0.639, 1.225)		0.461
History of any morbidity, past 14d	1.162 (0.796, 1.696)		0.438
Any ASF consumed in past 7d	1.124 (0.732, 1.725)		0.595
Chiefdom (vs. Jumbe)			
Mnkhanya	1.177 (0.751, 1.845)		0.476
Mwanya	1.082 (0.635, 1.843)		0.772
Nsefu	1.085 (0.612, 1.924)		0.780
*Between village (Level 2) variance*	*0*.*089*		
*ICC*	*0*.*026*		
*Overall R*^*2*^	*0*.*149*		

SES, socioeconomic status; BMI, body mass index; ICC, intraclass correlation coefficient

† Model includes fixed effects of Chiefdom and random effect of field site (village).

‡ Hierarchical livestock typology: Type 1 = no livestock; Type 2 = few poultry; Type 3 = many poultry; Type 4 = few mixed livestock; Type 5 = many mixed livestock

### Are more commonly used measures of livestock ownership associated with child nutrition outcomes?

We compared our findings using the typologies method with those using more traditional measures of livestock exposure. These analyses found that child DDS was negatively associated with any livestock ownership (β = -0.331, p = 0.002) or any chicken ownership (β = -0.320, p = 0.002; S2 Table). In both models, among livestock or chicken owners, increasing numbers of chickens, but not other animals, was positively associated with DDS (β = 0.016, p = 0.042 and β = 0.022, p = 0.009, respectively). No other measure of livestock ownership was significantly associated with DDS, ASF consumption, HAZ, or stunting (S2 Table). These results are strikingly similar to those found using livestock typologies as the sole measure of livestock ownership, which found a negative effect of livestock ownership on child DDS only among Type 2 livestock owners but not among those with larger livestock holdings or more valuable animals.

## Discussion

In this analysis, livestock ownership was not significantly associated with children’s odds of ASF consumption, HAZ, or odds of stunting in the Luangwa Valley. Furthermore, owning a small number of mostly poultry (Type 2) was actually associated with decreased overall child DDS compared to child DDS among households having no livestock (Type 1), an unexpected finding that diverges from traditional livestock development thinking. This finding was supported by additional analyses using more common measures of livestock ownership, which found that while owning any livestock was associated with significantly lower DDS, the association was almost entirely attributable to owning less than 15–20 chickens. This research reveals the complex association between livestock ownership and child nutrition outcomes in rural smallholder farming households and thereby helps lay the groundwork for the design of a livestock development programs that can optimize the impact on child diets and nutritional status in the Luangwa Valley. It additionally highlights the value of using a typologies approach as a proxy for how people use their livestock to uncover the differential and nuanced impact of various types and numbers of livestock on child nutrition outcomes.

Our data do not support the hypothesis that ownership of livestock is *necessarily* associated with greater ASF consumption among children. Because total livestock holdings in this population were very small on average, with a median TLU of just 0.05 (equivalent to five chickens), the slaughtering of animals for home consumption is likely very rare in most households, giving children few opportunities to benefit from livestock through more frequent meat consumption. Indeed, although the most commonly owned livestock were chickens, previous research has found that households here were reluctant to slaughter them for home consumption, preferring to sell chickens to pay school fees or cover emergency expenses [22]. That research also found that households rarely consumed eggs from village chickens, instead allowing them to hatch to increase flock sizes [22]. Similarly, consumption of goat, sheep, or cow’s milk produced by the household was extremely uncommon in this study. Therefore, in general, livestock were not kept by this population for routine ASF consumption at home, which is consistent with our finding that livestock ownership was not associated with higher ASF consumption or greater DDS.

Because we did not collect data on household income and expenditures, women’s empowerment, or crop yields, we cannot determine if traditional livestock ownership was associated with the other potential intermediate outcomes outlined in Fig 1. However, our analyses did not reveal an association between traditional livestock ownership and higher HAZ or lower odds of stunting in this population. We therefore conclude that if livestock did positively impact these unmeasured intermediate outcomes, the effect was: 1) too small to significantly improve child linear growth; 2) negated by negative consequences of livestock ownership; or 3) dwarfed by other factors responsible for child stunting (e.g. high prevalence of recent illness). Although not examined in this study, there are several reports that livestock ownership negatively affects child nutrition through increased pathogen exposure [16,20,32–36], increased maternal labor demands [37,38], or premature introduction of ASF to children [39]. Our finding of a very high incidence of child morbidity additionally suggests that overall health might be constraining any potential positive impact of livestock ownership.

Several methodological features of our current work provide insights that can help in intervention design and in monitoring and evaluation. First, our approach differed from several previous studies investigating the link between livestock ownership and child nutrition by categorizing total livestock holdings into five distinct typologies of livestock ownership. This approach allowed us to examine the differential impact of, for example, one chicken versus 15 chickens, or 15 chickens versus 15 goats, nuances which are lost when using binary or absolute count measures of livestock exposure. For example, had a single binary indicator been used as the only measure of livestock ownership, we would have concluded that livestock ownership was negatively associated with child DDS (β = -0.331; p<0.002; S2 Table). Upon closer examination, however, we see that this association can be accounted for almost entirely by ownership of chickens (β = -0.320; p<0.002). Furthermore, the negative effect of owning chickens decreases with each additional chicken owned (β = 0.022, p = 0.009), such that flocks greater with more than 15 chickens are no longer negatively associated with DDS. The typologies methodology therefore proved more efficient than an approach that tested multiple measures of livestock ownership individually and arrived at the same conclusion, while avoiding potential concerns of multicollinearity, which would prevent counts of multiple species from being considered in a single regression. In areas that are not dominated by holdings of a single particular species, our new methodology combining metrics might prove more informative and adaptive to different study sites, in which different typologies might need to be defined to reflect local environmental and cultural contexts.

Second, the majority of similar studies have been in a population of children under 5 years (with the exception of Nicholson et al. [12], which studied children under 6 years, and Gross [10], which studied only children 2–5 years), and only a minority disaggregated their results by child age. Given that almost all stunting occurs from conception to 24 months of age, this approach assumes that–for the older children in the study–a household’s livestock holdings did not change significantly over the two to three year period preceding measurement. By studying children 6–36 months, we have limited the lag from the time of meaningful exposure to the measurement of outcomes.

Finally, the context of our study site provides potential importance to people studying issues of “One Health,” a paradigm that explores the interconnected health relationships between people, domestic animals, and the environment [40]. The Luangwa Valley is a prime example of how these interactions operate in “buffer zones” around protected wildlife areas [21], where livestock production can be limited by predation and endemic infectious diseases at the wildlife-livestock interface [41,42]. As a result, fish and wildlife populations remain an important source of ASF for many communities living around protected areas [43,44], which may diminish the importance of domestic animals as a source of ASF, and instead encourage their use as sources of income [22]. Indeed, in this study, children living in Mwanya had the greatest dietary diversity and odds of ASF consumption, despite their low livestock ownership, high relative poverty (43.4% of households were categorized in the poorest tertile of the study population), and very poor market access and infrastructure. This, combined with anecdotal and published evidence, suggests a high dietary dependence on fish and wildlife population, which may therefore reduce the need for livestock to provide ASF [21]. Human populations living around similar protected areas throughout Africa are growing rapidly [45] and this study is among the first to examine the role of livestock production on child diets and nutrition within this unique context. Our results suggest that livestock-focused public health, development, and wildlife conservation programs operating in these areas should include nutrition behavior change communication and other program elements if they intend to positively impact child nutrition.

There are also some limitations to this analysis that should be considered when interpreting our results. First, both project and control communities were purposively selected to participate in the primary study based on their relationships with COMACO. We controlled for this non-probability based sampling strategy in our regression models, using random-effects variables to capture unobserved community factors and fixed-effects variables to control for observed household, maternal, and child characteristics. However, there are other factors that were either unmeasured (e.g. maternal education and self-efficacy, profession, ethnic group) or were largely unobservable within the context of our surveys (e.g. livestock management abilities, exposure to shocks such as catastrophic medical issues or idiosyncratic crop loss in the preceding year), that could modify or confound the relationship between livestock ownership and child outcomes. Second, because these data were not collected with the primary objective of evaluating the association between livestock ownership and child nutrition, we were only able to quantitatively evaluate one of the many hypothesized pathways in this relationship (i.e. ASF consumption). Additional data are necessary to consider all of the theoretical pathways linking livestock to child nutrition outcomes outlined in Fig 1. Third, the cross-sectional nature of this analysis prevents us from appreciating any temporal components to the relationship between livestock ownership and child nutrition outcomes. For example, a family currently owning livestock may have only recently acquired it, meaning the child has not had the opportunity to benefit from (or be harmed by) those livestock. Conversely, a family owning relatively few chickens might have previously had more birds but been forced to sell by a recent economic or medical shock. Children in that circumstance might have less evidence of stunting because of prior household conditions, but have lower DDS because of their current situation. A longitudinal study would better capture and quantify a child’s “lifetime exposure” to livestock ownership from conception. Finally, our findings are specific to the unique characteristics of this population in the Luangwa Valley and are not necessarily generalizable to other populations or settings.

These limitations notwithstanding, this research contributes to the growing body of literature suggesting that the link between livestock production and child nutrition outcomes is complex and likely highly context-specific. Livestock distribution is a common component of many rural development programs operated by charitable organizations aiming to improve income and/or ASF consumption. Our findings suggest, however, that simply *owning* livestock does not directly improve child diets or nutrition status in all situations.

From a policy perspective, this finding by no means implies that investments in poultry or other livestock cannot have a positive effect on child nutrition outcomes. On the contrary, analyses of some livestock development programs have found positive effects of dairy cooperatives, training, and technologies [46,47] and distribution of dairy cows or goats [48,49] on children’s diets or anthropometry. However, others have reported limited or no impact of other livestock development programs on nutrition outcomes [50–53]. This may in part be due to implementation failure, but our research additionally suggests that limited impact can be caused by a flawed program theory that assumes livestock ownership *necessarily* translates into improved diet and nutrition outcomes in any given context, an assumption that is challenged by our results. Development organizations must therefore carefully consider how their target population traditionally uses livestock to develop a theoretical framework identifying the likely links between livestock ownership and nutrition outcomes that is specific to that population and setting. Additionally, they should integrate ancillary elements into their program package, including nutrition and hygiene behavior change communication, training in optimal management practices, providing access to markets for ASF, and/or providing access to veterinary services. In these ways, they might ensure that *ownership* of livestock translates into actual nutritional benefits for children.

## Supporting information

S1 Table Summary of previous observational research on the link between livestock ownership and child nutrition outcomes in sub-Saharan Africa. (DOCX)

S2 Table Multilevel mixed-effects linear and logistic regression models assessing the relationship between four child nutrition outcomes and commonly used measures of livestock ownership. (DOCX)

S3 Table Variances and model diagnostics from null and adjusted models for the four outcomes of interest. (DOCX)

S1 Appendix Household, women’s, and children’s questionnaires. (PDF)
