# Supplementary material for: Examining the association between livestock ownership typologies and child nutrition in the Luangwa Valley, Zambia
Source: PLoS One. 2018 Feb 6;13(2):e0191339. doi: 10.1371/journal.pone.0191339 (PMC5800575; doi:10.1371/journal.pone.0191339)
Supplement: S1 Table — (DOCX) [file pone.0191339.s001.docx]

**S1 Table. Summary of previous observational research on the link between livestock ownership and child nutrition outcomes in sub-Saharan Africa.**

| Authors (Year), Country [Ref.] | Population age (n) | Measure of livestock | Outcomes | Findings |
| --- | --- | --- | --- | --- |
| Fierstein et al. (2017), Uganda [9] | 0 – 5 yr  (n= 2214) | - Binary measures of individual species (native cattle, nonnative cattle, equines, goats, sheep, pigs, and chickens) | HAZ | - Nonnative cattle ownership was positively associated with HAZ in rural children 0 – 5, *not* mediated by child dairy consumption - Sheep ownership was positively associated with HAZ in rural children 2 – 5 yr - Goat ownership was positively associated with HAZ in rural children 0 – 2 yr - In urban areas, only nonnative cattle ownership was associated with HAZ, and only in children 2 – 5 yr |
| Grosse (1999), Rwanda [10] | 24 – 59 mo  (n= 542) | - 3-level hierarchical index of dairy livestock (none, goats only, cows +/- goats) - Binary measure of poultry | HAZ | - Dairy animal ownership, but not poultry ownership, is associated with child HAZ |
| Hoddinott et al. (2015), Ethiopia [11] | 6 – 60 mo  (n= 4479) | - Binary measure of any cattle ownership | - Dairy product consumption (past 7d) - HAZ and stunting | - Cattle ownership increases likelihood and frequency of dairy consumption - Cattle ownership increases HAZ and reduced likelihood of stunting, with the greatest effect among children 12-18mo |
| Nicholson et al. (2003), Kenya [12] | 0 – 72 mo (n= 152) | - Binary measure of any cattle ownership - Count of dairy cows | - HAZ and WHZ | - Any cattle ownership (binary) is positively associated with HAZ, but not WHZ - The number of dairy cows owned is positively associated with HAZ, but not WHZ, and only in one of two regions |
| Okike et al. (2005), Ethiopia [13] | 0 – 60 mo  (n= 170) | - Tropical Livestock Units (TLU) - Count of cattle owned | - Child morbidities (past 14d) - HAZ | - TLU and number of cows are positively associated with child morbidity - Number of cows is positively associated with HAZ (association with TLU not reported) |
| Azzarri et al. (2015), Uganda [14] | 0 – 60 mo  (n= 3803) | - Individual count of livestock species | - Household ASF consumption - Probability of stunting, wasting, and underweight | - The numbers of poultry and large ruminants are positively associated with per capita household chicken and dairy consumption, respectively - Livestock ownership is not associated with stunting - Ownership of small ruminants is associated with a decreased probability of being wasted or underweight in children 2-5y; for underweight, this effect is partially countered by ownership of large ruminants |
| Iannotti & Lesorogol (2014), Kenya [15] | 0 – 18 yr  (n= 229) | - Individual counts of cattle, goats, and sheep owned | - Milk intake (24hr recall)   HAZ, WAZ, and BMIZ | - Goat and cattle, but not sheep, ownership increased household milk consumption - Milk consumption was not predictive of any anthropometric measure - Cattle ownership increased WAZ, but not HAZ, among children < 5y |
| Mosites et al. (2016), Kenya [16] | 1 – 60 mo  (n= 838) | - Total livestock count - Livestock count by species | - HAZ and WHZ - Annualized growth rate | Livestock ownership was not associated with HAZ, WHZ, or annualized growth rate |
| Jin and Iannotti (2014), Kenya [17] | 6 – 60 mo  (n= 183) | - Self-reported value of livestock by gender of owner | - ASF intake (7d recall) - HAZ, WAZ, and WHZ - Stunting, underweight, and wasting | - Co-/female-owned, but not male-owned, livestock was significantly associated with child ASF intake, HAZ, and WAZ - Child ASF intake mediated 25% of the effect from co/female-owned livestock on WAZ |
| Mosites et al. (2015), Ethiopia, Kenya, and Uganda [18] | 0 – 5 yr  (n, Ethiopia = 8079; n, Kenya = 3903; n, Uganda = 1645) | - Count of all animals - Individual counts of livestock species   TLU score | Stunting prevalence | - Total animal count was associated with slightly lower stunting prevalences in Ethiopia and Uganda, but not Kenya - TLU was not significantly associated with stunting prevalence in any country - Individual counts of cattle, goats, sheep, or chickens were not associated with stunting prevalence in any country - ASF intake did not modify effect between livestock and stunting prevalence |
| Good (2009), Ethiopia [19] | 6 – 18 mo (n= 297) | - Any small livestock production (sheep, goats, or chickens) | - ASF intake (60d recall) - Stunting, wasting, and underweight | - Children in households with small livestock consumed more eggs, but less cow’s milk, than those without - Children in households with small livestock were more likely to be stunted and underweight |
| Headey and Hirvonen (2016), Ethiopia [20] | 0 – 59 mo  (n= 3494) | - Binary measure of any poultry - Binary measure of other livestock | - HAZ - Egg and meat consumption (past 24 hr) | - Poultry ownership is positively associated with HAZ, but effect is negated by practice of corralling poultry in family home overnight - Other livestock ownership was not associated with HAZ - Poultry ownership is associated with increased egg, but not meat, consumption |

*Abbreviations*: ASF, animal source food; BMIZ, body mass index z-score; HAZ; height-for-age z-score; TLU, tropical livestock units; WAZ, weight-for-age z-score; WHZ, weight-for-height z-score
