# Supplementary material for: Examining the association between livestock ownership typologies and child nutrition in the Luangwa Valley, Zambia
Source: PLoS One. 2018 Feb 6;13(2):e0191339. doi: 10.1371/journal.pone.0191339 (PMC5800575; doi:10.1371/journal.pone.0191339)
Supplement: S2 Table — (DOCX) [file pone.0191339.s002.docx]

**S2 Table. Multilevel mixed-effects linear and logistic regression models assessing the relationship between four child nutrition outcomes and eleven commonly used measures of livestock ownership, compared with findings using livestock typologies as the measure of livestock ownership.**

| Model | Outcome | | | |
| --- | --- | --- | --- | --- |
|  | **Child DDS**  Adjusted β (95% CI) | **Odds of ASF consumption**  Adjusted OR (95% CI) | **HAZ**  Adjusted β (95% CI) | **Stunting odds**  Adjusted OR (95% CI) |
| Any livestock  (yes/ no) | **-0.331*****  (-0.542, -0.120) | 1.113  (0.730, 1.697) | 0.061  (-0.134, 0.259) | 0.857  (0.604, 1.217) |
| Total number of livestock | -0.003  (-0.012, 0.005) | 1.015  (0.994, 1.037) | -0.002  (-0.010, 0.007) | 1.005  (0.990, 1.020) |
| TLU | 0.003  (-0.062, 0.068) | 1.028  (0.895, 1.181) | -0.006  (-0.066, 0.053) | 1.030  (0.923, 1.150) |
| Any chickens | **-0.320*****  (-0.524, -0.116) | 1.084  (0.717, 1.638) | 0.052  (-0.140, 0.243) | 0.917  (0.653, 1.288) |
| Number of chickens (among chicken owners) | **0.022*****  (0.006, 0.039) | 1.023  (0.979, 1.069) | 0.006  (-0.010, 0.023) | 0.993  (0.963, 1.025) |
| Any goats | -0.130  (-0.455, 0.195) | 1.336  (0.676, 2.642) | -0.242  (-0.533, 0.050) | 1.201  (0.709, 2.035) |
| Number of goats  (among goat owners) | 0.004  (-0.037, 0.044) | 0.992  (0.857, 1.148) | -0.038  (-0.084, 0.008) | 1.044  (0.943, 1.155) |
| Any pigs | -0.244  (-0.567, 0.080) | 0.970  (0.501, 1.879) | -0.039  (-0.335, 0.258) | 1.085  (0.639, 1.841) |
| Number of pigs  (among pig owners) | 0.011  (-0.017, 0.039) | 0.996  (0.906, 1.094) | -0.021  (-0.060, 0.017) | 1.048  (0.960, 1.145) |
| Any cattle | 0.206  (-0.213, 0.626) | 1.815  (0.688, 4.787) | -0.065  (-0.444, 0.314) | 0.941  (0.464, 1.912) |
| Number of cattle (among cattle owners) | 0.126  (-0.009, 0.260) | 0.895  (0.568, 1.410) | 0.091*  (-0.007, 0.188) | 0.863  (0.641, 1.162) |
| Livestock typology (vs. Type 1) | |  |  |  |
| Type 2 | **-0.477*****  (-0.733, -0.221) | 0.976  (0.582, 1.636) | 0.206*  (-0.025, 0.437) | 0.683*  (0.445, 1.048) |
| Type 3 | -0.216  (-0.612, 0.179) | 0.932  (0.419, 2.075) | 0.036  (-0.315, 0.379) | 0.7530  (0.387, 1.463) |
| Type 4 | -0.318  (-0.744, 0.106) | 0.682  (0.313, 1.488) | -0.324*  (-0.703, 0.055) | 1.404  (0.708, 2.781) |
| Type 5 | -0.180  (-0.447, 0.087) | 1.782*  (0.990, 3.207) | -0.034  (-0.276, 0.207) | 1.031  (0.659, 1.612) |

All models included field site random effects nested within Chiefdom to control for geographic clustering, as well as controls for households, maternal, and child characteristics. ASF, animal source foods; DDS, dietary diversity score; HAZ, height-for-age z-score; TLU, tropical livestock units

* p< 0.1; ** p< 0.05; *** p< 0.01
