# Supplementary material for: Examining the association between livestock ownership typologies and child nutrition in the Luangwa Valley, Zambia
Source: PLoS One. 2018 Feb 6;13(2):e0191339. doi: 10.1371/journal.pone.0191339 (PMC5800575; doi:10.1371/journal.pone.0191339)
Supplement: S3 Table — (DOCX) [file pone.0191339.s003.docx]

**S3 Table. Variances and model diagnostics from null and adjusted models for the four outcomes of interest.**

|  | **Dietary Diversity** | | **ASF consumption** | | **HAZ** | | **Stunting** | |
| --- | --- | --- | --- | --- | --- | --- | --- | --- |
|  | **Null** | **Adjusted** | **Null** | **Adjusted** | **Null** | **Adjusted** | **Null** | **Adjusted** |
| **Random effects†** |  |  |  |  |  |  |  |  |
| Level 2 variance | 0.056 | 0.029 | 0.325 | 0.176 | 5.28E-17 | 3.52E-17 | 0.049 | 0.089 |
| Level 1 variance | 2.062 | 1.823 |  |  | 1.688 | 1.452 |  |  |
| **Fit statistics** |  |  |  |  |  |  |  |  |
| -2 log likelihood | -1453.0 | -1340.0 | -394.4 | -343.8 | -1342.9 | -1282.6 | -561.5 | -485.7 |
| Wald chi2 |  | 113.9 |  | 68.28 |  | 130.2 |  | 86.18 |
| AIC | 2912 | 2840 | 793 | 723 | 2692 | 2613 | 1127 | 1017 |
| ICC (rho) | 0.027 | 0.016 | 0.090 | 0.051 |  | 0.000 | 0.015 | 0.026 |
| Overall R^2^**‡** |  | 0.1256 |  | 0.1380 |  | 0.1398 |  | 0.1490 |
| R^2^, Level 2 |  | 0.4821 |  |  |  | 0.3333 |  |  |
| R^2^, Level 1 |  | 0.1159 |  |  |  | 0.1398 |  |  |

AIC, Akaike’s information criterion; ICC, intraclass correlation coefficient; ASF, animal source food; HAZ, height-for-age z-score

**†** Level 2 variances refers to the between village variance; Level 1 variance refers to the within village (between household) variance. Stata does not estimate Level 1 variance for random-intercept logistic models.

**‡** For binary outcome, R^2^ was calculated using the method described in Tjur, T. (2009) “Coefficients of determination in logistic regression models—A new proposal: The coefficient of discrimination.” *The American Statistician* 63: 366-372. For continuous outcomes, R^2^ and its composite parts calculated from Raudenbush & Bryk (2002) *Hierarchical Linear Models: Applications and Data Analysis Methods.* 2^nd^ ed. Thousand Oaks, CA: Sage.
