# Supplementary material for: Examining the association between livestock ownership typologies and child nutrition in the Luangwa Valley, Zambia
Source: PLoS One. 2018 Feb 6;13(2):e0191339. doi: 10.1371/journal.pone.0191339 (PMC5800575; doi:10.1371/journal.pone.0191339)
Supplement: S1 Appendix — (PDF) [file pone.0191339.s004.pdf]

Associated Field Site code: ____________________________HH code: ___________________________

Household Food Security and Wealth Survey, Form 1 DEC 2014

Administered to head of household

Section 1. Household demographics

A. Location details

1.1 *Date:* ____ / _____ /______ *Start time:* ________________

1.2 *Interviewer:* _______________________________________

1.3 *Chiefdom:* ________________________ **1.4** *GPS of HH:* ______________________________

1.5 *VAG:*____________________________ **1.6** Village: _________________________________

**1.7**	Is anyone in this household (HH) a member of COMACO? *(0= no; 1= yes)*	
**1.8**	Is any member of this HH involved in the operation of the egg layer facility? *(0= no; 1= yes; 88= NA, not applicable if in control site)*	

B. Household details

1.11 Is the head of household a man or woman? *(1= male, 2= female)* ________

1.12 Now I want to ask you about the people living in your household. By 'household', I mean people who live together with you and with whom you share meals at least 4 days per week. This may include your spouse, children, adopted children, parents and in-laws, other extended family, and servants. Do not count children away at school or living elsewhere. Please give first names only.

Note: <u>For children 5 years or less, month and year of birth must be recorded and exact age in months must be calculated!</u> *For anyone over 5 years, only one of YEAR BORN or AGE needs to be completed for each individual, not both. Use whichever method respondent prefers. For adults, their 'best guess' is acceptable, or enter 'DK' for 'don't know'. DK cannot be used for children. Use comments box for additional people if more than 12 people reside in this HH.*

a) First, what is the first name of the head of household? What is your age, or, if it is easier, what year where you born? Did you ever attend school? What is the highest year of education that you completed?
b) Are you married? What is the name of the first wife who is living in this household? What is (SPOUSE)'s age, or the year of her birth? Did she ever attend school? What is the highest year of education that she completed? *Continue for all spouses residing in this HH.*
c) What is the name of your oldest child that lives in this HH? Is (CHILD) a boy or a girl? What is (CHILD)'s age or year of birth? *If less than 5 years:* What is the month and year of (CHILD)'s birth? Is (CHILD) in school? *If yes*: What grade is (CHILD) in? *If no:* Has (CHILD) ever been in school? What is the highest year of education that (CHILD) completed? *Continue for all children residing in HH.*
d) Is there anyone else that lives in this household and with whom you share meals? *If yes:* What is this person's name? Is (NAME) a male or female? What is (NAME)'s relation to you? What is the year of (NAME)'s birth, or (NAME)'s age? Is (NAME) in school? What is (his/her) current grade or the highest year of education completed? *Continue for all people in household.*
e) So there are (NUMBER OF PEOPLE) living in this household. Is that correct? Is there anyone else who lives and eats meals in this HH?

Note: <u>Circle all children ages 6-36 months who are eligible for Form 3 and Form 4.</u>

[Individual Code] Name	Relation to HH head *(options below)*	Month / Year born (*MM/YYYY*) **OR**	Age *(circle mos or yrs)*	Sex (*1=male, 2=female)*	Education *(codes below)* In school?	Current or highest education
[1]	Head of HH	/	yrs mos		a)	b)
[2]		/	yrs mos		a)	b)
[3]		/	yrs mos		a)	b)
[4]		/	yrs mos		a)	b)
[5]		/	yrs mos		a)	b)
[6]		/	yrs mos		a)	b)
[7]		/	yrs mos		a)	b)
[8]		/	yrs mos		a)	b)
[9]		/	yrs mos		a)	b)
[10]		/	yrs mos		a)	b)
[11]		/	yrs mos		a)	b)
[12]		/	yrs mos		a)	b)

Relation to HH head: *spouse (married or cohabitating), son/daughter, son/daughter-in-law, grandchild, mother/father, mother/father-in-law, brother/sister, brother/sister-in-law, uncle/aunt, nephew/niece, step/foster/adopted child, other family, not related*

Codes, education: *a) In school: 0=no; 1= yes; 88=NA, less than 5 years; b) Current or highest level of education: 0= none; 1= some primary, less than grade 4; 2= some primary, completed at least grade 4; 3= completed primary; 4= some secondary; 5= completed secondary; 6= beyond secondary; 88= NA, less than 5 years; 99= don't know*

Section 2. Household wealth proxies

C. House

**2.1**	What type of material are (most of) the walls of the house made from? (*1= reeds/ straw/ grass/ bamboo; 2= mud/ soil; 3= wood; 4= iron/metal; 5= bricks/ concrete; 77= other, specify)*	
**2.2**	What type of material is (most of) the roof of the house made from? (*1= thatch; 2= wooden; 3= iron/metal; 4= tiles; 77= other, specify)*	
**2.3**	What type of material is (most of) the floor of the house made from? (*1= earth/ mud/ soil; 2= concrete/ cement; 3= tiles/ vinyl; 77= other, specify)*	
**2.4**	How many total rooms are in the house? *(enter number of rooms)*	
**2.5**	What is the household's main method of cooking food? *(1= fire; 2= charcoal cooker; 3= high-efficiency cook stove; 4= solar cooker; 5= electric or gas stove; 77= other, specify)*	
**2.6**	Does your household have access to any type of electricity, including solar power? (*0= none; 1= solar panel; 2= connected to grid; 3= battery (non-solar); 77= other, specify)*	

D. Water and sanitation

**2.7**	What is the main source of drinking water for the household? (*1= open source/ surface water; 2= unprotected dug well; 3= protected dug well; 4= borehole; 5= public tap/ standpipe; 6= private piped water; 77=other source, specify*)	
**2.8**	What type of toilet does the family use? (*1= no toilet/ open/ bush; 2= bucket; 3= shared pit toilet/latrine; 4= private pit toilet/latrine; 5= flush/pour toilet; 77= other, specify*)	

E. Assets: Does any member of the household own (ITEM NAME)?

Item		NO= 0; YES= 1	Item		NO= 0; YES= 1
**2.9**	TV		**2.21**	Wooden cart or wheelbarrow	
**2.10**	Radio		**2.22**	Gun	
**2.11**	Cassette/ CD/ VHS/ DVD player		**2.23**	Snare, for hunting	
**2.12**	Refrigerator/ freezer		**2.24**	Bed	
**2.13**	Car/ truck		**2.25**	Sofa	
**2.14**	Motorcycle		**2.26**	Table	
**2.15**	Bicycle		**2.27**	Treadle water pump	
**2.16**	Mobile phone *(see Q 1.8)*		**2.28**	Solar panel	
**2.17**	Boat		**2.29**	Invertor or battery	
**2.18**	Plough		**2.30**	Clock/ Watch	
**2.19**	Mattress		**2.31**	A bank or savings account	
**2.20**	Agricultural land that is worked by members of your household?		**2.32**	Other item worth more than K160, specify: _____________	

F. Hired labor

**2.31**	Is there anyone who works in your household for money or food, but is unrelated to any member of the family? *(0=no; 1= yes)*	

Section 3. Household income sources

G. Income from crops and livestock

**3.1**	Do any members of this household own or rent any agricultural land, including small gardens, or bee-hives/ apiaries? *(0= no, SKIP to Q 3.3; 1= yes)*	

3.2 What types of crops do you grow? ***Note:*** *Create a list of the crops farmed by the household. Then, for each crop, ask parts (a), (b) and (c). Probe for honey production and garden vegetables.*

**Crop Type**	(a) Does your household eat the (CROP TYPE) that you grow? (*0= no; 1= yes; 88= NA*)	(b) Do you ever sell, barter or trade the (CROP TYPE) that you grow for cash or other goods? (*0=no; 1= yes, sometimes; 2=yes, regularly*)

**3.3**	Does any member of this household own livestock of any kind, including cattle, goats, pigs, chickens, farmed fish, or other types of livestock? *(0= no, SKIP to Q 3.5; 1= yes)*	

3.4 What types of livestock do you own? ***Note:*** *Create a list of livestock types. Then, for each type, ask parts (a) and (b). Probe for poultry, farmed fish, and other livestock types listed in your manual.*

**Livestock Type**	(a) What is the main reason that your household raises (LIVESTOCK TYPE)? (*See codes below; use up to two codes for each livestock type)*		(b) Do you ever sell, barter or trade the (LIVESTOCK TYPE) or its products for cash or other goods? *(0= never; 1= sometimes; 2= regularly)*	(c) How many of this (LIVESTOCK TYPE) does the household have at this moment?

Codes (a): *1= family consumption of animals; 2= family consumption of animal products (milk or eggs); 3= consumption on special occasions (to serve to visitors, celebration, holiday); 4= to sell animals; 5= to sell animal products (milk or eggs); 6= for fertilizer; 7= to prepare land; 77= other, specify*

H. Income from natural resources: Does anyone in this household sell (ITEM NAME)?

**3.5**	Timber, firewood, or charcoal? *(0=no; 1= yes)*	
**3.6**	Wild-caught (non-farmed) fish? *(0=no; 1= yes)*	
**3.7**	Other wild products including wild nuts, seeds, berries, mushrooms, game animals, wild honey, and/or medicinal plants? *(0=no; 1= yes)*	

I. Compensation and wages

**3.8**	Has any member of the HH had paid work over the past 6 months, including during the planting or harvesting season, such as piece-work or casual labor? Paid work may include working for compensation in kind (i.e. for meali meal). This does not include income from a business that this household owns. *(0= no, SKIP to Q 3.11; 1= yes)*	
**3.9**	Has any member of the household had paid work over the past 30 days, such as piece-work or casual labor? Paid work may include working for compensation in kind (i.e. for meali meal). This does not include income from a business that this household owns. *(0= no, SKIP to Q 3.11; 1= yes)*	

3.10 What type of work has (he/she) done? How many days over the past 30 days did (he/she) do this work? *Probe:* Any other paid work in the HH? ***Note:*** *One person can be listed more than once for different jobs.*

**HH member (using individual code from 1.12)**	**Type of work**	**Days worked in past 30 days**

J. Income from non-agriculture business

**3.11**	Has anyone in this household received income from his/her own business over the past 30 days, not including the sources of income already mentioned above? *(0= no, SKIP to Q 3.13; 1= yes)*	

3.12 What type of business does (he/she) have? *Probe:* Any other business income in the household?
Note: *Confirm that business has not already been listed in parts G, H, and I before listing it here.*

HH member (using individual code from 1.12)	Type of business

K. Other income sources (*use as many codes as applies)*

**3.13** Are there any other sources of income received by the household over the past 3 months?						

Codes: *0= no other sources of income; 1= regular income from a family member living and working elsewhere; 2= gifts or financial support from relatives or friends; 3= cash support from government, church, or other organization; 4= pension; 5= payment for renting out land; 77= other, specify*

Section 4. Household food security

L. Household Food Insecurity Access Scale (HFIAS): The next set of questions is about your household's access to food over the past 4 weeks. ***Note:*** *"Rarely"= once or twice in the past 4 weeks; "Sometimes" = 3 to 10 times in the past 4 weeks; "Often"= more than 10 times in the past 4 weeks*

Question	Response options	Code
**4.1** In the past 4 weeks, was there ever a time that you were worried that your household would not have enough food?	*0= No (SKIP to Q 4.3)*<br>*1= Yes*	
**4.2** How often did this happen?	*1= Rarely; 2= Sometimes; 3= Often*	
**4.3** In the past 4 weeks, was there ever a time that you or any household member were not able to eat the kinds of foods you preferred because of a lack of resources?	*0= No (SKIP to Q 4.5)*<br>*1= Yes*	
**4.4** How often did this happen?	*1= Rarely; 2= Sometimes; 3= Often*	
**4.5** In the past 4 weeks, was there ever a time that you or any household member had to eat a limited variety of foods due to a lack of resources?	*0= No (SKIP to Q 4.7)*<br>*1= Yes*	
**4.6** How often did this happen?	*1= Rarely; 2= Sometimes; 3= Often*	
**4.7** In the past 4 weeks, was there ever a time that you or any household member had to eat some foods that you really did not want to eat because of a lack of resources to obtain other types of food?	*0= No (SKIP to Q 4.9)*<br>*1= Yes*	
**4.8** How often did this happen?	*1= Rarely; 2= Sometimes; 3= Often*	
**4.9** In the past 4 weeks, was there ever a time that you or any household member had to eat a smaller meal than you felt you needed because there was not enough food?	*0= No (SKIP to Q 4.11)*<br>*1= Yes*	
**4.10** How often did this happen?	*1= Rarely; 2= Sometimes; 3= Often*	
**4.11** In the past 4 weeks, was there ever a time that you or any household member had to eat fewer meals in a day because there was not enough food?	*0= No (SKIP to Q 4.13)*<br>*1= Yes*	
**4.12** How often did this happen?	*1= Rarely; 2= Sometimes; 3= Often*	

**4.13**	In the past 4 weeks, was there ever at time when there was <u>no food to eat of any kind in your household</u> because of lack of resources to get food?	*0= No (SKIP to Q 4.15)*<br>*1= Yes*	
**4.14**	How often did this happen?	*1= Rarely; 2= Sometimes; 3= Often*	
**4.15**	In the past 4 weeks, was there ever a time that you or any household member <u>went to sleep at night hungry</u> because there was not enough food?	*0= No (SKIP to Q 4.17)*<br>*1= Yes*	
**4.16**	How often did this happen?	*1= Rarely; 2= Sometimes; 3= Often*	
**4.17**	In the past 4 weeks, was there ever a time you or any household member <u>went a whole day and night without eating anything</u> because there was not enough food?	*0= No (SKIP to Part M)*<br>*1= Yes*	
**4.18**	How often did this happen?	*1= Rarely; 2= Sometimes; 3= Often*	
		**Total HFIAS Score (Range 0 – 27):**	

M. Coping Strategies Index (CSI): These last questions ask you about the things that you did when your household did not have enough food or money to buy food in the past <u>7 days</u>.

Note: *Ask part (a) first. If the answer is "yes", probe with part (b). If the answer is "no", write 0 in (b).*

**a) In the past 7 days, when you did not have enough food or money to buy food, has your household ever had to (ROW ITEM)?**	**b) In the past 7 days, how many days did this happen?** *(enter 0 - 7)*
**4.19** Rely on less preferred and less expensive foods	
**4.20** Borrow food or money, or rely on help from a friend or relative	
**4.21** Purchase food on credit	
**4.22** Gather wild plants for food	
**4.23** Hunt with a gun or snare	
**4.24** Harvest immature crops	
**4.25** Consume seed stock being held for next season	
**4.26** Send household members to eat elsewhere	
**4.27** Send household members to beg	
**4.28** Limit portion size at mealtimes	
**4.29** Cook differently in order to spread the meal among more people	
**4.30** Decrease your own meal size in order for children to eat	
**4.31** Limit food for non-working members of the household to feed working members	
**4.32** Replace a meal with tea, water or porridge	
**4.33** Reduce number of meals eaten in a day	
**4.34** Go entire days without eating	

N. Months of Adequate Household Food Provisioning (MAHFP)

**5.1**	Think back over the past 12 months, all the way to December/January of last year. Were there months, in the past 12 months, when you did not have enough food to meet your family's needs? *(0= no; 1= yes)*	
**5.2**	*If yes:* Which were the months in the past 12 months during which you did not have enough food to meet your family's needs?<br>[ ] January [ ] February [ ] March [ ] April [ ] May [ ] June [ ] July<br>[ ] August [ ] September [ ] October [ ] November [ ] December	
**5.3**	Total number of months with inadequate foods: _______	

Finish time: ________________

Women's Nutrition and Wellbeing Survey, Form 2 DEC 2014

Administered to woman 15-49 years old

Section 1. Interview details

1.1 *Date:* ____ / _____ /______ *Start time:* ________________

1.2 *Interviewer:* _______________________________________

**1.3** Woman's first name: _________________________	**1.4** *Code (Q1.12, Form 1):*_________

**1.5** Are you currently pregnant? *(0= no, 1= yes, 99= don't know)*	
**1.6** *If yes:* What trimester in your pregnancy are you in? *(1= first; 2= second; 3= third, 88= NA, not pregnant; 99= don't know)*	
**1.7** Are you currently breastfeeding? *(0= no, 1= yes)*	
**1.8** How old is the child you are breastfeeding? *(enter in months)*	
**1.9** What is your marital status? *(1= married or living with a man as if married; 2= single; 3= separated; 4= widowed; 5= divorced; 6= have a boyfriend who lives separately)*	
**1.10** *If married or cohabitating:* Including yourself, how many wives or partners does your husband have?	
**1.11** *If >1 wife*: Are you the first, second, third, …, wife? *(enter rank)*	

Section 2. Women's Dietary Diversity Score (WDDS)

2.1 How many meals did you eat yesterday during the day or night? ____________

Please describe all foods, meals, and snacks that you ate or drank yesterday during the day and night. Start with the first food or drink of the morning. ***Note:*** *Write down all foods and drinks mentioned. When mixed dishes are mentioned, ask for a list of ingredients and list them.*

Morning	Afternoon	Evening

- *When the recall is complete, circle each food that is mentioned in the 'Example Items' column below and place a '1' in each row if any items from that food group were mentioned*
- *If the food mentioned is not listed below, circle it above and discuss with supervisor.*
- *Ignore any foods eaten in small amounts (< 1 tablespoon), like seasoning*
- *For any food group not mentioned, probe:* (Yesterday/ 2 days ago) during the day or night, did you eat any (FOOD GROUP), such as (EXAMPLE ITEMS)?

	**Food groups**	**Example items**	**NO= 0 YES= 1**
**2.2**	Starchy staples (cereals, roots, tubers)	Maize, rice, wheat, sorghum, millet, other grains or foods made from grains (nshima, bread, buns, fritters, noodles, porridge); Irish potatoes, white yam, white sweet potatoes, cassava, manioc, other foods made from roots	
**2.3**	Dark green leafy vegetables	Kale, rape, amaranth, cassava leaves, Chinese rape, cowpea leaves, spinach, collard greens, pumpkin leaves (chiwawa), sweet potato leaves, other leafy greens, including wild forms	
**2.4**	Vitamin A rich fruits and veggies	Pumpkin, butternut, carrot, squash, red or yellow yams, orange, red or yellow sweet potato, red sweet pepper; mango, cantaloupe, apricot, papaya (paw paw), peach	

**2.5**	Other fruits and vegetables	Other vegetables not included above (tomato, onion, eggplant, cabbage, mushrooms, green peppers, okra, cucumber); other fruits not mentioned above (wild fruit, bananas, oranges, guava, grapes, masau, watermelon, baobab, cashew nut fruit, nchenja, African horned melon (kiwano, orange spiky cucumber))	
**2.6**	Organ meat	Liver, kidney, heart or other organ meats (offal)	
**2.7**	Meat and fish	Beef, pork, lamb, goat, rabbit, bushmeat, nyama, field mice, chicken, duck, other birds, insects or grubs, caterpillars, fresh or dried fish, kapenta	
**2.8**	Eggs	Eggs from chicken, duck, guinea fowl, or any other egg	
**2.9**	Legumes, nuts and seeds	Beans, soybeans, cowpeas, green gram (kankhoma), pigeon peas, peas, lentils, groundnuts, sunflower seeds, sesame, other nuts, seeds or foods made from these (e.g. peanut butter, soy milk, Soya pieces)	
**2.10**	Milk products	Milk, cheese, yogurt, or other milk products from animal source	
		**Total WDDS (add 2.2 through 2.10; range 0–9):**	

**2.11**	In the past 30 days, have you eaten anything that is not food because you have cravings (or strong desire) for it, like earth/soil, clay, stones, ash, charcoal, or uncooked rice, uncooked flour, or uncooked potatoes? *(0=no; 1=yes)*	

In the past 7 days, how many times did you eat (ROW ITEM)?		**No. of times**
**2.12**	Meat of any kind (e.g. beef, pork, nyama/bushmeat, lamb, goat, chicken, duck, guinea fowl), but not including fish or kapenta	
**2.13**	Fish	
**2.14**	Kapenta	
**2.15**	Milk, cheese, yogurt, or other milk products from animal source	
**2.16**	Eggs from chicken	
**2.17**	Eggs from other birds, like duck, guinea fowl, or any other non-chicken poultry	
	**Total ASF Score (add 2.12 through 2.17):**	

What is the main reason that you don't eat more:		**Code** *(see below)*
**2.18**	Meat of any kind, but not fish or kapenta	
**2.19**	Fish	
**2.20**	Kapenta	
**2.21**	Milk	
**2.22**	Eggs of any kind	

Codes: *1= cost, it is too expensive; 2= lack of availability, it is difficult to find; 3= I don't like or prefer not to eat it; 4= I'm allergic; 5= religion/ traditional beliefs; 6= someone else in the HH does not eat it; 77= other, explain; 88= NA, we eat as much as we want and need*

When you do eat (ITEM), where do you mostly get it from?		**Code** *(see below)*
**2.23**	Meat of any kind, but not fish or kapenta	
**2.24**	Fish	
**2.25**	Kapenta	
**2.26**	Milk	

Codes: *1= livestock raised at home; 2= purchased from market or road-side stalls; 3= purchased from family or neighbors; 4= borrowed/gifted from family or neighbors; 5= hunting/fishing from wild; 77= other, explain; 88= NA, we don't eat it*

Section 3. Food decisions and perceptions

**3.1.**	Who is usually served first at meals in your household? *(1= Woman herself; 2= spouse; 3= children; 4= other adult men (not husband); 5= other adult women (not woman herself); 6= all served at the same time, SKIP next two questions; 77= other, specify*	
**3.2.**	Who is usually served second at meals in your household? *(1= Woman herself; 2= spouse; 3= children; 4= other adult men (not husband); 5= other adult women (not woman herself); 6= all served at the same time; 77= other, specify*	
**3.3.**	Who is usually served last at meals in your household? *(1= Woman herself; 2= spouse; 3= children; 4= other adult men (not husband); 5= other adult women (not woman herself); 6= all served at the same time; 77= other, specify*	

**3.4.**	Who usually decides what types of foods the family will eat? *(1= woman herself; 2= husband; 3= husband and wife are equally responsible for deciding; 4= mother or father; 77= other, specify)*	

Egg preference and consumption

**3.5**	Do you like eggs? *(0= no; 1= yes)*	
**3.6**	What are the main reasons that you and your family eat eggs? (*1= easy/quick; 2= low cost; 3= taste/ we like them; 4= nutritious; 5= change the diet; 77= other, specify)*	
**3.7**	In the past 7 days, how many chicken eggs did this HH eat in total?	
**3.8**	In the past 7 days, how many eggs from other birds did this HH eat in total?	

Egg handling

**3.9**	After bringing eggs into the HH, how long do you usually store them before you eat them? (*0= eat them immediately, otherwise enter number of days)*	
**3.10**	Before cooking eggs, do you wash the outside of them? (*0= no; 1= yes)*	
**3.11**	Do you usually buy eggs raw or already cooked (hardboiled)? (*1= raw; 2= already cooked (hardboiled))*	
**3.12**	There are many ways to cook eggs. How do you usually cook eggs? (*1= scrambled; 2= hardboiled; 3= fried; 4= mixed with other relish; 5= in a baked food (e.g. cake); 77= other, specify ______________)*	

Perception about egg safety and social acceptability

Show Image 1, the 5 point scale of agreement level. For the next set of questions, I will read you some about eggs. You can choose to strongly disagree, mildly disagree, neither agree nor disagree, mildly agree, or strongly agree. You can see in the pictures of this woman's face are meant to represent each of these feelings, each associated with a number. For each statement, choose a number or point to a face that most closely matches how you feel about the statement. Remember, there is no right answer!

**3.13**	Eggs are safe to eat.	1= strongly disagree to 5= strongly agree	
**3.14**	Raw or undercooked eggs can make me sick.	1= strongly disagree to 5= strongly agree	
**3.15**	Eggs that are too old are more likely to make me sick than eggs that are fresh.	1= strongly disagree to 5= strongly agree	
**3.16**	I should wash my hands after handling raw eggs.	1= strongly disagree to 5= strongly agree	
**3.17**	There is more risk of getting sick from eating Soya Pieces or vegetables than from eating eggs.	1= strongly disagree to 5= strongly agree	
**3.18**	The eggs that are available to buy in this area are fresh and safe to eat.	1= strongly disagree to 5= strongly agree	
**3.19**	Eggs available to buy at the shop or at road-side stalls are not as safe as eggs taken directly from a hen.	1= strongly disagree to 5= strongly agree	
**3.20**	Eggs taken directly from the hen have more nutrients than eggs bought at the shop or road-side stalls.	1= strongly disagree to 5= strongly agree	
**3.21**	Eggs should be stored in a cool place to keep them fresh and safe.	1= strongly disagree to 5= strongly agree	
**3.22**	Eggs are good for small babies (under 12 months) to eat.	1= strongly disagree to 5= strongly agree	
**3.23**	Eggs are good for toddlers (12 months to 3 years) to eat.	1= strongly disagree to 5= strongly agree	
**3.24**	Eggs are good for school age children to eat.	1= strongly disagree to 5= strongly agree	
**3.25**	Eggs are good for pregnant women to eat.	1= strongly disagree to 5= strongly agree	

**3.26**	Eggs are good for breastfeeding women to eat.	1= strongly disagree to 5= strongly agree	

**3.27**	Are there any people who are not supposed to eat eggs because of traditional or cultural reasons? *(0= no, SKIP to 3.21; 1= yes)*	
**3.28**	Who is not supposed to eat eggs? *(1= babies, 2= young children, 3= pregnant women, 4= breastfeeding women, 5= any women, 77= other, specify)*	

Physical accessibility of eggs

**3.29**	Where does the HH get most of the eggs that it eats? (*1= own village hens; 2= road-side stalls; 3= store; 4= own layer hens/ production facility; 5= nearby egg production facility; 6= neighbor's village hens; 77= other, specify)*	
**3.30**	How long do you usually travel to get eggs? *(enter number of minutes)*	

Section 4. Women's health and wellbeing

Subjective wellbeing. *Show respondent Image 2, "The Ladder"*

**4.1**	Please imagine a ladder with steps numbered from 1 at the bottom to 10 at the top, like the one in this picture. The top of the ladder represents the best possible life for you (10) and the bottom of the ladder represents the worst possible life for you (0). Please point to the step of the ladder that you personally feel that you stand at this time?	
**4.2**	Using your best guess, what step do you think you stood on in the past, around 2009?	
**4.3**	Using your best guess, what step do you think you will stand on in the future, say in 2019?	

Physical and emotional health. Let's look at the ladder again. This time, for each question I will tell you what the lowest and highest steps on the ladder mean, and you can point to any step or tell me any number from 0 to 10 that represents how you would like to answer the question. Please feel free to ask for clarifications as we go. ***Note:*** *As you define the steps, point to the lowest step as you read (0) and the highest step as you read (10).*

		**Step definitions**	**Code**
**4.4**	Overall, how would you rate your health during the past 4 weeks?	0= very poor health<br>10= excellent health	
**4.5**	During the past 4 weeks, did you have any difficulty doing your daily work, either at home or away from home, because of physical health problems?	0= no (enter '10' for Q4.6 and SKIP to Q4.7)<br>1= yes (go to Q4.6)	
**4.6**	*If yes:* Would you rate this difficulty that you had doing your daily work as extreme difficulty (0), moderate difficulty (5), small difficulty (9), or somewhere in between?	0= you were not able to work at all<br>10= you were able to do everything without limitations	
**4.7**	During the past 4 weeks, did you have any bodily pain?	0= no (enter '10' for Q4.8 and SKIP to Q4.9)<br>1= yes (go to Q4.8)	
**4.8**	*If yes:* Would you rate this pain as the worst possible pain (0), moderate pain (5), very low pain (9), or somewhere in between?	0= very severe pain<br>10= no pain at all	
**4.9**	During the past 4 weeks, how much energy did you have? Almost no energy (0), moderate energy (5), very good energy (10), or somewhere in between?	0= no energy<br>10= very good energy	
**4.10**	During the past 4 weeks, have you been feeling anxious, sad, depressed, or irritated at all?	0= no (enter '10' for Q4.11 and SKIP to Q4.12)<br>1= yes (go to Q4.11)	

**4.11**	*If yes:* Did you have this feeling of anxiety, sadness or depression all the time (0), about half the time (5), just a few times (9), or somewhere in between?	0= all the time<br>10= not at all	
**4.12**	During the past 4 weeks, did your physical health or emotional problems limit your usual social activities with family or friends at all?	0= no (enter '10' for Q4.13 and SKIP to Q4.14)<br>1= yes (go to Q4.13)	
**4.13**	*If yes:* How much did your health problems limit your social activities? Did it completely limit you from doing any social activities (0), limit you somewhat (5), limit you just a little bit (9), or somewhere in between?	0= you have not been able to do any social activities<br>10= you have not been limited in your social activities at all	

Finish time: ________________

Associated Field Site code: ________________________ HH code: ___________________________

<u>Child Nutrition Survey, Form 3</u> DEC 2014

Administered to mother of selected child, or primary caregiver of child if mother is not in household

Section 1. Interview details

1.1 *Date:* ____ / _____ /______ *Start time:* ________________

1.2 *Interviewer:* ____

1.3 Respondent's first name: ___________________________________

**1.4** Child's first name: _______________________	***1.5*** *Child's code (Q1.12, Form 1):*_______

1.6 Child's date of birth (MM/YYYY): ________ *Calculate child's age:* _________*months*

Section 2. Child's Dietary Diversity Score (CDDS).

2.1 How many meals did (CHILD) eat yesterday during the day and night? _______

I would like you to describe everything that (CHILD) ate yesterday during the day and night, both at home and outside the home. ***Note:*** *Write down all foods and drinks mentioned. When mixed dishes are mentioned, ask for a list of ingredients and write them out separately.*

a) When (CHILD) first woke up yesterday, did (he/she) eat anything at that time? *If yes:* Please tell me everything (CHILD) ate at that time. *Probe:* Anything else? *Continue probe until nothing else.*
b) What did (CHILD) do after that? Did (CHILD) eat anything at that time? *If yes:* Please tell me everything (CHILD) ate at that time. *Probe:* Anything else? *Continue probe until nothing else.*

Repeat question (b) above until respondent says that the child went to sleep for the night.

c) Did (CHILD) sleep through the night? *If no:* Did (CHILD) eat anything during the night?

Morning	Afternoon	Evening / Night

- *Probe for <u>condiments, sugar, and oils/fats.</u>*
- *When recall is complete, circle each food that is mentioned in 'Example Items' column below and place a '1' in each row if any items from that food group were mentioned.*
- *If the food mentioned is not listed below, circle it above and discuss with supervisor*
- *If foods are used in small amounts for seasoning, include them as "condiments"*

For any food group not mentioned, probe: (Yesterday/ 2 days ago) during the day or night, did (CHILD) taste and (FOOD GROUP), such as (EXAMPLE ITEMS)?

	**Food groups**	**Example items**	**NO= 0 YES= 1**
**2.2**	Starchy staples (cereals)	Maize, rice, wheat, sorghum, millet, other grains or foods made from grains (nshima, bread, buns, fritters, noodles, porridge)	
**2.3**	Vitamin A rich vegetables	Pumpkin, butternut, carrot, squash, red or yellow yams, red or orange or yellow sweet potato, red sweet pepper	
**2.4**	Roots and tubers	Irish potatoes, white yams, white sweet potato, manioc, cassava, other roots or tubers	
**2.5**	Dark green leafy vegetables	Kale, rape, amaranth, cassava leaves, Chinese rape, cowpea leaves, spinach, pumpkin leaves (chiwawa), collard greens, sweet potato leaves, other leafy greens, including wild forms	
**2.6**	Vitamin A rich fruits	Ripe mango, cantaloupe, apricot, papaya (paw paw), peach	
**2.7**	Other fruits and vegetables	Other vegetables not included above (e.g. tomato, onion, eggplant, cabbage, mushrooms, green peppers, okra, cucumber); other fruits not mentioned above, including wild fruit, bananas, oranges, guava, grapes, masau, watermelon, baobab, nchenja, cashew nut fruit, African Horned melon (kiwano, orange spiky cucumber)	
**2.8**	Organ meat	Liver, kidney, heart or other organ meats (offal)	
**2.9**	Meat (excluding fish)	Beef, pork, lamb, goat, rabbit, bushmeat (nyama), field mice, chicken, duck, pigeon, other poultry; not including insects or grubs	
**2.10**	Eggs	Eggs from chicken, duck, guinea fowl, or any other egg	
**2.11**	Fish	Fresh or dried fish, kapenta, shellfish	
**2.12**	Legumes, nuts and seeds	Beans, soybeans, cowpeas, green gram (kankhoma), pigeon peas, peas, lentils, sunflower seeds, groundnuts, sesame, other nuts, seeds or foods made from these (e.g. peanut butter, Soya Pieces)	
**2.13**	Milk and milk products	Animal-sourced milk, cheese, yogurt, cream, or other milk products; NOT INCLUDING BREASTMILK	
**2.14**	Oils and fats	Any oil, fats, butter, or foods made with any of these	
**2.15**	Sugar	Any sugary foods, e.g. sugar, sugar cane, chocolate, sweets, candy, pastries, cakes or biscuits, honey, soft-drink, juice or juice drinks	
**2.16**	Condiments	Anything used in small amounts, including chilies, spices, herbs, fish powder, salt, baking soda (including spices in Soya Pieces)	
**2.17**	Insects	Grubs, snails, caterpillars, or insects	
		**Total CDDS (add 2.2 through 2.17; range 0–16):**	

In the past 7 days, how many times did (CHILD) eat (ROW ITEM)?		**No. of times**
**2.18**	Meat of any kind (nyama/ bushmeat, field mice, beef, pork, lamb, goat, chicken, duck, guinea fowl), but not including fish or kapenta	
**2.19**	Fish	
**2.20**	Kapenta	
**2.21**	Milk, cheese, yogurt, or other animal-sourced milk products (NOT INCLUDING BREASTMILK)	
**2.22**	Eggs from chicken	
**2.23**	Eggs from duck, guinea fowl, or any other non-chicken poultry	
	**Total ASF Score (add 2.18 through 2.23):**	

Section 3. Breastfeeding history

**3.1**	Has (CHILD) ever been breastfed? *(0= no, SKIP to Q 3.4; 1= yes; 99= don't know)*	
**3.2**	Was (CHILD) breastfed yesterday during the day or night? (*0= no; 1= yes, SKIP to 3.4; 99= don't know)*	
**3.3**	At what age did you stop breastfeeding (CHILD)? (*Enter in months. Enter '0' if less than 1 month)*	
**3.4**	Sometimes babies are fed breast milk in different ways, by spoon, cup or bottle, or are breastfed by another woman. Did (CHILD) consume breast milk in any of these ways yesterday during the day or night? (*0= no, SKIP to 3.7; 1= yes; 99= don't know)*	
**3.5**	Was (CHILD) given breast milk in this way yesterday? *(0= no; 1= yes, SKIP to 3.7)*	
**3.6**	At what age did you stop giving (CHILD) breast milk in this way? *(Enter in months)*	
**3.7**	At what age did (CHILD) drink water for the first time? (*Enter in months. Enter '0' if less than 1 month.)*	
**3.8**	At what age did (CHILD) eat solid or semi-solid foods for the first time? (*Enter in months. Enter '0' if less than 1 month.)*	

For children 24 months of age or less:

Section 4. Liquids

Did (CHILD) have any (ROW ITEM) yesterday during the day or night?
If yes for 4.2, 4.3 or 4.5: How many times yesterday during the day or night did (CHILD) consume (ITEM)?

**Item**		**0= NO; 1= YES; 99= DON'T KNOW**	**No. of times**
**4.1**	Plain water?		
**4.2**	Infant formula?		
**4.3**	Milk from cow, goat, or other animal (non-breast milk, incl. powdered, tinned, or fresh)?		
**4.4**	Juice or juice drinks?		
**4.5**	Clear broths?		
**4.6**	Very thin porridge?		
**4.7**	Other liquids? (specify: ________________________)		

For all children:

Section 5. Child health

Did (CHILD) have (ROW ITEM) in the past 2 weeks?		**0= NO; 1= YES; 99= DON'T KNOW**
**5.1**	Diarrhea (≥ 3 stools in one day)?	
**5.2.**	Diarrhea with blood in the stool?	
**5.3.**	Fever?	
**5.4.**	Illness with cough and short, rapid breathing?	
**5.5.**	Diagnosis of malaria by a health professional?	
**5.6.**	Vomiting	

Finish time: ________________

Associated Field Site code: ______________________ HH code: ________________________

Luangwa Valley Anthropometrics, Form 4

For all children in HH aged 6 to 36 months of age and their mothers (age 15-49 years), or other woman completing Form 2

Section 1. Session details Date: ____ / _____ / ______

Measurer 1: ________________________________ Measurer 2: ________________________________

Section 2. Child anthropometric data. *Check Question 1.12 on Form 1 for any children age 6 to 36 months in this HH. Enter names and codes from Form 1, Q1.12. Confirm age in months. Enter the mother's individual code or check "NA" if the mother is deceased or otherwise not a member of the household. Take a third measure if the difference between the first two measures is greater than or equal to the allowed difference. Check for bilateral edema.* ***Ref= refused; NA= not applicable (if third measure is not needed)***

	Child 1	Child 2	Child 3	Child 4
**2.1** Child name / code				
**2.2** Child's age	________ months	________ months	________ months	________ months
**2.3** Mother's code	______ ☐ NA	______ ☐ NA	______ ☐ NA	______ ☐ NA
**2.4 Weight (kg)**				
First measure	______. ___ kg	______. ___ kg	______. ___ kg	______. ___ kg
Second measure	______. ___ kg	______. ___ kg	______. ___ kg	______. ___ kg
Third measure (*if differences ≥ **0.5 kg)***	______. ___ kg	______. ___ kg	______. ___ kg	______. ___ kg
**2.5 Height (cm)**				
First measure	_______. ___ cm	_______. ___ cm	_______. ___ cm	_______. ___ cm
Second measure	_______. ___ cm	_______. ___ cm	_______. ___ cm	_______. ___ cm
Third measure (*if difference ≥ **1 cm)***	_______. ___ cm	_______. ___ cm	_______. ___ cm	_______. ___ cm
**2.6** Measured lying down or standing up?	☐ Lying down<br>☐ Standing up	☐ Lying down<br>☐ Standing up	☐ Lying down<br>☐ Standing up	☐ Lying down<br>☐ Standing up
**2.7 MUAC (cm)**				
First measure	______. ___ cm	______. ___ cm	______. ___ cm	______. ___ cm
Second measure	______. ___ cm	______. ___ cm	______. ___ cm	______. ___ cm
Third measure (*if difference ≥ **0.5 cm)***	______. ___ cm	______. ___ cm	______. ___ cm	______. ___ cm
**2.8 Bilateral edema?**	☐ Yes ☐ No	☐ Yes ☐ No	☐ Yes ☐ No	☐ Yes ☐ No

Section 3. Maternal anthropometric data. *Measure all women age 15-49 years with a child measured above. If mother is not currently at home, make a callback appointment. If the mother of child is deceased, or if no eligible child is in the HH but the HH is part of the egg production group, measure one woman age 15-49 years who completed Form 2.* ***Ref= refused; NA= not applicable (if third measure is not needed)***

	Woman 1	Woman 2	Woman 3
**3.1** Woman name / code			
**3.2 Weight (kg)**			
First measure	________. ___ kg	________. ___ kg	________. ___ kg
Second measure	________. ___ kg	________. ___ kg	________. ___ kg
Third measure (*if differences ≥ **0.5 kg)***	________. ___ kg	________. ___ kg	________. ___ kg
**3.3 Height (cm)**			
First measure	_______. ___ cm	_______. ___ cm	_______. ___ cm
Second measure	_______. ___ cm	_______. ___ cm	_______. ___ cm
Third measure (*if difference ≥ **1cm)***	_______. ___ cm	_______. ___ cm	_______. ___ cm
**3.4 MUAC (cm)**			
First measure	______. ___ cm	______. ___ cm	______. ___ cm
Second measure	______. ___ cm	______. ___ cm	______. ___ cm
Third measure (*if difference ≥ **0.5 cm)***	______. ___ cm	______. ___ cm	______. ___ cm
